# Exploiting EST databases for the development and characterisation of 3425 gene-tagged CISP markers in biofuel crop sugarcane and their transferability in cereals and orphan tropical grasses

**DOI:** 10.1186/1756-0500-6-47

**Published:** 2013-02-04

**Authors:** Amaresh Chandra, Radha Jain, Sushil Solomon, Shiksha Shrivastava, Ajoy K Roy

**Affiliations:** 1 Division of Plant Physiology and Biochemistry, Indian Institute of Sugarcane Research, Rae Bareli Road, 226002, Lucknow, Uttar Pradesh, India; 2 Indian Grassland and Fodder Research Institute, Gwalior Road, 284003, Jhansi, Uttar Pradesh, India

**Keywords:** Conserved-intron scanning primers, Sugarcane, Tropical grasses, Oat, Gene annotation, Gene-tagged markers

## Abstract

**Background:**

Sugarcane is an important cash crop, providing 70% of the global raw sugar as well as raw material for biofuel production. Genetic analysis is hindered in sugarcane because of its large and complex polyploid genome and lack of sufficiently informative gene-tagged markers. Modern genomics has produced large amount of ESTs, which can be exploited to develop molecular markers based on comparative analysis with EST datasets of related crops and whole rice genome sequence, and accentuate their cross-technical functionality in orphan crops like tropical grasses.

**Findings:**

Utilising 246,180 *Saccharum officinarum* EST sequences *vis*-*à*-*vis* its comparative analysis with ESTs of sorghum and barley and the whole rice genome sequence, we have developed 3425 novel gene-tagged markers — namely, conserved-intron scanning primers (CISP) — using the web program GeMprospector. Rice orthologue annotation results indicated homology of 1096 sequences with expressed proteins, 491 with hypothetical proteins. The remaining 1838 were miscellaneous in nature. A total of 367 primer-pairs were tested in diverse panel of samples. The data indicate amplification of 41% polymorphic bands leading to 0.52 PIC and 3.50 MI with a set of sugarcane varieties and *Saccharum* species. In addition, a moderate technical functionality of a set of such markers with orphan tropical grasses (22%) and fodder cum cereal oat (33%) is observed.

**Conclusions:**

Developed gene-tagged CISP markers exhibited considerable technical functionality with varieties of sugarcane and unexplored species of tropical grasses. These markers would thus be particularly useful in identifying the economical traits in sugarcane and developing conservation strategies for orphan tropical grasses.

## Background

Sugarcane (*Saccharum* spp. hybrids) is an important commercial crop, grown between 30^0^ N and 30^0^S. Approximately 70 and >35% of the world’s sugar [1] and alcohol ( [2], OECD-FAO Agricultural Outlook 2011–2020) production, respectively, comes from sugarcane. It is a genetically complex polyploid grass, belonging to the family Poaceae and to the tribe Andropogoneae, like maize and sorghum. Modern sugarcane varieties are mainly derived from interspecific crosses between the noble cane *Saccharum officinarum* (2n = 80) and the wild species *S. spontaneum* (2n = 40–128). The complexity of the sugarcane genome largely associated with polyploidy/aneuploid nature and variation in chromosome number [3], has hindered the genetic analysis even though a reasonable level of microsatellite or simple sequence repeat (SSR) markers have been developed and reported [2,4-9].

Contrary to research in important cereal crops, the progress in sugarcane genomics is considerably slow. Nevertheless, the key turning points in the recent past, apart from the development of SSR markers, have been: better understanding of the evolutionary origin and genome structure; development and accumulation of important resources such as genetic maps, large ESTs ( http://sucest-fun.org/en/projects/sucest-fun/sucest-fun-database); and availability of BAC library.

The complete genome sequence of rice can be seen as a boon for the comparative analysis of those crop genomes where sufficient ESTs are available, as in sugarcane. The fact that maize and sorghum are closely related grasses can further speed up the work on sugarcane. Any novel gene-based markers for a case like sugarcane, having a large genome not amenable to complete sequencing, shall always be in demand for various purposes, including varietal identification, genetic diversity and phylogenetic studies, quantitative trait loci (QTL) identification and association mapping [10-15]. Four species of tropical grasses, namely, *Dichanthium annulatum*, *Sehima nervosum*, *Heteropogon contortus* and *Chrysopogon fulvus*, are major component of grasslands of tropical regions of Australia and Asia (mainly the Indian sub-continent). However, these species have not yet been considered in developing EST sequences and diversity analysis utilising gene-tagged markers. Some of the promising genotypes of *D. annulatum* have been analysed using isozyme, RAPD and ISSR markers [16-19]. Most of these grasses are apomictic in nature; however, they exhibit wide diversity because of facultative sexuality. The characterisation of germplasm is required to maintain identity and purity, for proper conservation and management *vis-à-vis* identifying unique lines as they grow unabatedly under harsh environmental conditions. In India, a collection of >750 accessions (collected from different parts of the country) of these four grasses is maintained at Indian Grassland and Fodder Research Institute (IGFRI), Jhansi, but so far these grasses have not been characterised molecularly.

Both genomic and EST-SSR markers are marred by limitations. Genomic SSRs are mostly developed from the non-coding regions and do not provide information about the synteny or function of the coding section of the genome. On the other hand, EST-SSRs have detected low levels of polymorphism among clones of *Saccharum* species and related genera [20]. Only 1515 gSSRs [5,9] and 342 EST-SSRs [8] have been available for assessing agronomically important traits in the complex sugarcane genome. Considering sugarcane’s large genome size, these values are much smaller. Thus, there is a need for rapid alternative methods for their development, having both high polymorphism and transferability. Sequencing and detailed functional analysis of the Poaceae genomes, such as rice [21], maize [22] and sorghum [23] has opened new avenues in comparative biology, providing for the improvement of many complex crops including sugarcane. This approach has already been successfully utilised for deciphering the sugarcane genetics associated with brown-rust resistance [24].

For efficient application of genomic tools across taxa for genetic-diversity study, there is a need to identify conserved genomic sequences as well as variation at the DNA level [25]. Based on this understanding, a new marker system, conserved-intron scanning primers (CISP), was developed in different crop plants [26-28]. In CISPs, relatively conserved exons, located near exon–intron boundaries, are used to scan introns for suitably variable markers [27]. Large EST sequences available for a crop like sugarcane, when aligned with rice genomic sequences, primers allow amplification of genomic DNA across intron regions, producing PCR products that exhibit polymorphism either due to size or presence/absence of DNA fragments. These gene-based markers can identify genes inexpensively and have been used successfully in identifying polymorphic markers in legumes, pearl millet and other grasses [26,27,29]. Fredslund *et al*. [26] designed and developed 76 CISP markers, of which, 56 (73.7%) and 43 (56.6%) markers were tested for cross-species amplification in bean and peanut, respectively. It has also been reported that CISPs are an effective means to explore poorly characterised genomes, for both, DNA polymorphism and non-coding sequence conservation on a genome-wide or candidate-gene basis, and to provide anchor points for comparative genomics, across a diverse range of species [30]. Recently, Zeid *et al*. [28] assessed the cross-transferability of CISPs among 16 grass species (including cereal, turf and forage crops) and found the highest amplification rate for CISPs developed from pearl millet (91.1%) and sorghum (75.1%) ESTs which, aligned with rice sequences. Recently, we reported 30 CISP markers in sugarcane, for the first time, showed a moderate level of polymorphism (55.2%). Mean polymorphism information content value was 0.582 and genetic-similarity coefficient ranged from 0.39 to 0.95, indicating variable levels of divergence depending on the taxonomic rank assessed [31]. In the present study, we report a total of 3425 CISP markers which will facilitate understanding of the extent of natural variation at molecular level. This in turn will help develop new strategies for sugarcane improvement program world wide and India in particular, as the genetic base of modern Indian sugarcane cultivars is considered narrow due to use of limited number of parental species clones in cross-hybridisation and repeated intercrossing of hybrids [14,32]. Also, sets of these gene-tagged markers were employed in assessing the genetic diversity of the accessions of four major tropical grasses as well as forage oat genotypes, to visualise the level of cross-amplification of CISP markers and assess the level of polymorphism.

## Materials and methods

### Plant materials and EST retrieval

Nineteen genotypes representing four species of *Saccharum* and commercial sugarcane cultivars, as well as 30 accessions of four major tropical grasses (namely *Dichanthium annulatum, Heteropogon contortus, Sehima nervosum* and *Chrysopogon fulvus*) and 6 accessions of *Avena sativa*, were used to see the technical functionality and investigate the genetic diversity and level of transferability of CISP markers developed, based on the EST sequences of sugarcane, barley, sorghum and whole genome sequence of rice. Most of the *Saccharum* and sugarcane cultivars are maintained and conserved at National Hybridisation Garden (NHG) at Coimbatore, India while tropical grasses and *Avena sativa* are conserved at a mid-term module located at Indian Grassland and Fodder Research Institute, Jhansi, India (Table 1). Major agronomical traits of sugarcane varieties and *Saccharum* species (namely, time of maturity, level of sugar in stalk, biotic and abiotic stress tolerance as well as most useful traits of tropical grasses and oat genotypes) are also given in Table 1.

**Table 1:** **List of genotypes belonging to *Saccharum *complex, tropical grasses and oat used in the present study**

**Sl. No.**	**Genus and species**	**Clones/ accessions/ varieties**	**Agronomical traits**	**Sl. No.**	**Genus and species**	**Clones/ accessions/ varieties**	**Agronomical traits**
**Commercial sugarcane varieties and *Saccharum *species**				29	*Heteropogon contortus*	IG-HC-1	MT,MTi,MG
1	*Saccharum* spp. hybrids	CoLk8102	MLM,MS,MRRR	30	*Heteropogon contortus*	IG-HC-2	T,HTi,VGG
2	*Saccharum* spp. hybrids	CoLk8001	EM,HS,MRRR	31	*Heteropogon contortus*	IG-HC-3	T,MTi,GG
3	*Saccharum* spp. hybrids	CoS767	MLM,HS, FT	32	*Heteropogon contortus*	IG-HC-4	T,MTi,GG
4	*Saccharum* spp. hybrids	CoLk9606	MLM,HS,MRRR	33	*Heteropogon contortus*	IG-HC-5	MT,LTi,PG
5	*Saccharum* spp. hybrids	CoLk9617	MLM,MS,RRS	34	*Dichanthium annulatum*	IG97-234	MT,HTi,,MG
6	*Saccharum* spp. hybrids	CoS95255	EM,MS,MRRR	35	*Dichanthium annulatum*	IG97-24	MT,MTi,PG
7	*Saccharum* spp. hybrids	BO91	MLM,MS,FT	36	*Dichanthium annulatum*	IG95-30	MT,HTi,GG
8	*Saccharum* spp. hybrids	CoJ64	EM,HS,MRRR	37	*Dichanthium annulatum*	IG97-192	MT,MTi,GG
9	*Saccharum* spp. hybrids	Co1148	MLM,HS,HTV	38	*Dichanthium annulatum*	IG97-247	MT,HTi,MG
10	*Saccharum* spp. hybrids	CoS97264	MLM,MS,RRR	39	*Dichanthium annulatum*	IG97-241	T,HTi,GG
11	*Saccharum* spp. hybrids	CoSe92423	MLM,MS,MRRR	40	*Dichanthium annulatum*	IG95-25	MT,LTi,PG
12	*Saccharum* spp. hybrids	CoLk94184	EM,HS,FT,MRRR	41	*Sehima nervosum*	IG-SN-1	T,HTi,VGG
13	*Saccharum spontaneum*	SES34	VLS,AST,PDR	42	*Sehima nervosum*	IG-SN-2	T,HTi,GG
14	*Saccharum spontaneum*	Coimbatore	VLS,AST,PDR	43	*Sehima nervosum*	IG-SN-3	T,LTi,MG
15	*Saccharum barberi*	Saretha	EM,MS,TnS,AST	44	*Sehima nervosum*	IG-SN-4	T,MTi,MG
16	*Saccharum officinarum*	28NG210	EM,HS,TkS,PDS	45	*Sehima nervosum*	IG-SN-5	VT,VHTi,VGG
17	*Saccharum sinense*	Kavenzire	EM,LMS,TnS	46	*Sehima nervosum*	IG-SN-6	T,LTi,GG
18	*Saccharum sinense*	Malani	EM,LMS,TnS	47	*Sehima nervosum*	IG-SN-7	MT,VHTi,GG
19	*Saccharum* spp. hybrids	BO138	MLM,HS,FT,MRRR	48	*Sehima nervosum*	IG-SN-8	T,HTi,GG
**Tropical grasses**				49	*Sehima nervosum*	IG-SN-9	MT,HTi,GG
20	*Chrysopogon fulvus*	IG-CF-1	T,MTi,GG	**Oat genotypes**					
21	*Chrysopogon fulvus*	IG-CF-2	VT,MTi,GG	50	*Avena sativa*	JHO 822	VT,MTi,MFl		
22	*Chrysopogon fulvus*	IG-CF-3	T,GTi,MG	51	*Avena sativa*	JHO 99-2	T,HTi,MFl		
23	*Chrysopogon fulvus*	IG-CF-4	MT,GTi,MG	52	*Avena sativa*	NGB 6370	MT,HTi,LFl,L		
24	*Chrysopogon fulvus*	IG-CF-5	MT,MTi,MG	53	*Avena sativa*	JHO 851	MT,HTi,MG,MFl,L		
25	*Chrysopogon fulvus*	IG-CF-6	MT,GTi,GG	54	*Avena sativa*	NGB 4871	MT,GTi,LFl,L		
26	*Chrysopogon fulvus*	IG-CF-7	T,GTi,VGG	55	*Avena sativa*	NGB 4462	MT,MTi,LFl,L		
27	*Chrysopogon fulvus*	IG-CF-8	MT,MTi,GG						
28	*Chrysopogon fulvus*	IG-CF-9	MT,MTi,MG						

Commercial sugarcane varieties and *Saccharum* species.

EM = Early maturing; MLM-Mid-late maturing, HS = High sugar; MS = Medium sugar; LMS = Low-medium sugar; VLS = Very low sugar; FT = Flood tolerant; RRR = Red-rot resistant; MRRR = Moderate red-rot resistant; RRS = Red-rot susceptible; AST = Abiotic stress tolerant; TkS = Thick stalk; TnS = Thin stalk; PDS = Pest and disease susceptible; PDR = Pest and disease resistant; HTV = High tillering variety.

Tropical grasses and Oat genotypes.

T = Tall; MT = Medium tall; VT = Very tall; LTI = Low tillering; MTI = Medium tillering; HTI = High tillering; VHTI = Very high tillering; GG = Good growth; MG = Medium growth; VGG = Very good growth; MFl = Medium flowering; LFl = Late flowering; L = Leafy.

*S. officinarum* EST sequences were downloaded from TIGR Plant Transcript Assemblies ( http://plantta.jcvi.org/index.shtml). A total of 246,180 ESTs derived from different tissues were used for the design of CISP markers. The FASTA-formatted files of EST sequences were downloaded for the purpose.

### Development of CISP primers

The polymorphism identification strategy, which focuses on introns of highly conserved genes, was taken into consideration for the identification of cross-species genetic marker candidates, as defined by sets of primer pairs for PCR amplification of introns. We used model plants like rice to predict intron positions in its cDNA/EST sequences and then design a pair of primers on both sides of each intron position. Thus, these specific primers would potentially detect intron length polymorphisms in the target plant sugarcane, as well. These cross-species CISPs were developed using GeMprospector program [26]. We used the Blast program package from NCBI for sequence comparisons with the cut-off *E*-value 10^-7^ for sequence homology. Clustalw ( http://www.ebi.ac.uk/Tools/msa/clustalw2/) was used to perform multiple alignments. The *S. officinarum* EST sequences were downloaded and compared with database of homologous ESTs clusters (from sorghum and barley) and genomic sequences of rice. Multiple sequence alignment among these sequences formed the basis of automated PCR primer design in conserved exons, in such a way that each primer set amplifies an intron. A total of 3425 CISP primers were designed and developed for the present work (see Additional file 1).

### DNA extraction, PCR amplification and sequence analysis

Fresh and young leaves from 13 commercial sugarcane cultivars and six genotypes representing four species of *Saccharum* were collected in liquid nitrogen, and DNA was isolated by the method described by Doyle and Doyle [33]. Genomic DNA from tropical grasses and *Avena sativa* plants was isolated following the procedure of Iqbal *et al.*[34], with minor modification earlier reported by us [16]. The quality and quantity of genomic DNA was checked on 0.7% agarose gel and the concentration of DNA was finally kept at 5 ng/μl for PCR amplification. Amplification was carried out in a 15 μl reaction mixture consisting of 1X PCR assay buffer (Bangalore Genei Pvt. Ltd., India), 200 μM of the four dNTPs (Bangalore Genei Pvt. Ltd., India), 10 μM each of forward and reverse primers (Imperial Life Sciences (P) Limited, India), 0.5 units of Taq DNA polymerase (Bangalore Genei Pvt. Ltd., India) and 25 ng template DNA. PCR reactions were carried out in a thermal cycler (PTC 200, Bio Rad, USA) using the following cycling parameters: initial denaturation at 94°C for 3 min, followed by 35 cycles at 94°C for 30 sec, 62°C for 30 sec, 72°C for 30 sec and finally a primer extension cycle of 10 min at 72°C. The amplified products were resolved in 3% high quality agarose gel made in 0.5X TBE buffer, at 70 V and visualised under UV light following staining with ethidium bromide (MERK, Germany). The band size of the amplicons generated by the CISP markers was determined using 100 bp DNA ladders (MBI Fermentas, Lithuania) as a size standard.

The amplified products (alleles) from varieties of sugarcane and species of *Saccharum* were eluted and cloned into pGEM-T Easy vector following the manufacturer’s instructions. After purification recombinant clones were directly sequenced using suitable primer with an automated sequencer ABI 3730XL (Applied Biosystems, Foster City, CA, USA). The online tool ClustalW2 ( http://www.ebi.ac.uk/Tools/msa/clustalw2/) was used to align the sequences.

### Polymorphism analysis and genetic-similarity estimate

The amplified products were scored for presence (1) or absence (0) of bands and data was entered in a binary data matrix, as discrete variables. Bands present in all genotypes were considered as monomorphic. The amplified fragments produced by the CISP were considered alleles of a single locus. The polymorphism information content (PIC) was calculated for each marker by applying the formula of Roldan-Ruize *et al.*[35]: PIC_*i*_ = 2*f*_*i*_ (1 − *f*_*i*_), where *f*_*i*_ is the frequency of the amplified allele (band present) and (1 − *f*_*i*_) is the frequency of the null allele (band absent) of marker *i*. Marker index (MI) was determined as the product of PIC and the number of polymorphic bands per assay unit [36].

The presence-absence matrix was constructed and used to estimate the genetic similarity between all the genotypes evaluated. Dice’s coefficient of similarity was calculated and a dendrogram was constructed by using Unweighted Pair Group Method of Arithmetic Mean Analysis (UPGMA). The computer package NTSYS-PC Version 2.02 [37] was used for cluster analysis. The same software was used to perform the Mantel’s test of correlation between the cophenetic values and the Dice’s similarity coefficients to ascertain reliability of the clusters obtained. The confidence limits of UPGMA based dendrogram was determined by bootstrap analysis. One thousand bootstrap replicates were computed and bootstrap of 50% majority rule consensus tree was constructed using the bootstrap procedure of the WinBoot software program [38].

## Findings

### Development of CISP markers and evaluation of their polymorphic potential in sugarcane and related species

**Figure 1:**
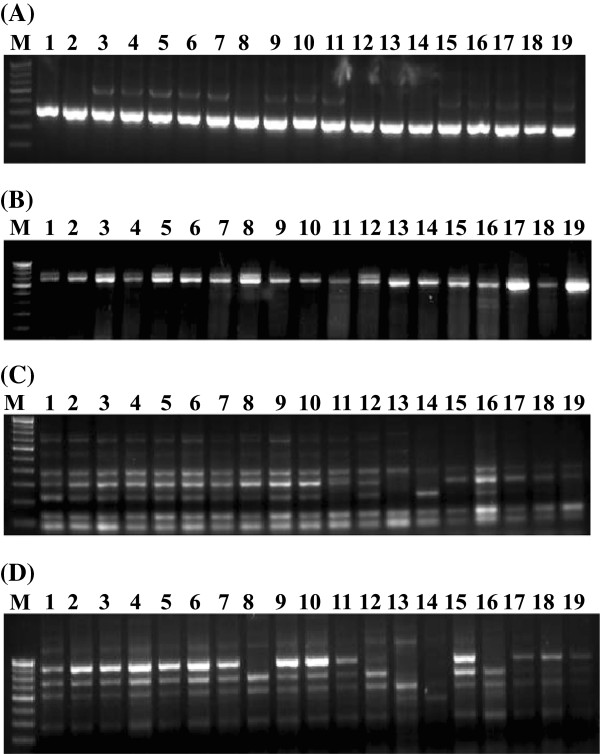
**DNA length polymorphism as observed with CISP markers.** PCR products and their length polymorphism of three CISP markers (CISP_SC-31 (**A**), CISP_SC-39 (**B**) CISP_SC-276 (**C**) and CISP_SC-348 (**D**) on 3% high quality agarose gel among 19 genotypes comprising 13 sugarcane varieties and six *Saccharum* species (see Table 1 for details). M = 100 bp DNA ladder as molecular marker.

A total of 3425 conserved intron scanning primers (CISP) were identified from 246,180 publicly available EST sequences (EST sequences were downloaded from TIGR Plant Transcript Assemblies site in March 2009) of *Saccharum officinarum*. By identifying the regions of sequence conservation across related species like sorghum, barley and introns of rice, primer pairs were designed in such a manner that the segment containing the intron is amplified. Using the rice genome sequence, the intron positions were precisely predicted and then a pair of primers on both sides of each intron position was designed. This has maximised the chance that the primers work for species like sugarcane where no sequence information is available. In addition, the PCR product contains polymorphism, making the locus a potential genetic marker. As per the built-in characteristics of the GeMprospector software, all indices were blasted against the rice genome and single-copy sequences were kept, indexed by their rice homologue. Relevant gene indices were compared against their genomes in order to identify sequences with rice introns. Gene indices are intron-tagged at the corresponding positions. Following these hypothesis, in total 3425 primer pairs (PPs) were developed and designed (See Additional file 1). Initially, we tested 30 PPs with DNA of sugarcane and related taxa and results indicated reactivity of 29 PPs [31]. In the present study, another group of 337 PPs (CISP_SC-31 to −367) based on their functional significance related to carbohydrate metabolism, photosynthesis-related proteins and many other traits, were tested with 13 sugarcane varieties released from different research stations of the country and tested with four species (namely, *S. spontaneum, S. barberi, S. officinarum* and *S. sinense*) (Table 1). The number of fragments amplified, PIC and other details are given in additional file 2. Of these 337 PPs, 185 (55%) have reacted with 19 DNA representing a panel of 13 sugarcane varieties and accessions of four species of *Saccharum* (Table 1). In total, 515 fragments were scored. Of these, 211 bands (41%) were polymorphic while the rest (59%) were monomorphic. The number of fragments varied from 1 to 9, with size ranging from 100 to 2500 bps (Figure 1). Only 16 PPs (4.7%) generated 6 or more bands, and the maximum number of bands (9) was obtained with CISP_SC-340 (Additional file 2). Of these 337 PPs, the gene sequences of many represent functional genes, including carbohydrate metabolism and photosynthesis-related genes (Additional file 2). Hence, the expected reactivity of these primers was in general high. However, the results obtained in the present study have rather shown a reasonably low level (55%) of reactivity with sugarcane varieties or *Saccharum* species. One possible reason could be the complex nature of ploidy existing in sugarcane and also the rearrangements of sequences in chromosomes, leading to the deterioration of varieties with time or rearrangements of specific genes during the evolution process. Chromosomal rearrangements have been reported in sugarcane genome. Sugarcane is also endowed with certain cytogenetic peculiarities, such as highly heterozygous polyploidy depicting 2n + n gametic transmission (instead of n + n) and en-bloc elimination of chromosomes during cell division. The high level of ploidy further complicates the situation, as individual genotypes will encompass multiple alleles at one locus, and loci are also likely to be duplicated. This could be the reason for the amplification of more than one fragment, though only those with 4.7% PPs multiple bands were observed. Even simple sequence repeat (SSR) markers, which are otherwise regarded as co-dominant markers, are treated as dominant markers in sugarcane, as it is seldom clear whether they represent unique alleles at a single locus or duplicated loci [39]. In the case of rice and *Rhododendron*, only 4–5% intron-flanking primers have generated multiple bands [40,41]. As suggested earlier, these multiple bands may arise due to paralogous sequences with high similarity in the genome or these multiple bands generating primer pairs span a region of introns with high variability [41].

In order to assess the functional significance of the CISP, gene ontology classification of the 3425 CISP primer pairs, developed on the basis of conserved sequences of sugarcane, sorghum, barley and intron of rice, resulted in three major categories. Of the 3425 that were functionally annotated, 1096 (32%) were encode expressed proteins, 491 (14%) were hypothetical proteins and the remaining 1838 (54%) were miscellaneous in nature (Additional file 1).

The molecular markers based on the comparative genomics have proved their utility for genetic improvement of grasses. The cross-species markers developed in the present study has the advantage of alignment of the merger of databases of the groups of the intra-homologous sequences, with the user data (i.e., sugarcane). Being a complex polyploid crop, sugarcane requires comparatively high polymorphic molecular markers resource for genetic studies. An initial effort has been made to find out the suitability of CISP markers in sugarcane.

### Evaluation of genetic relationships among germplasm

**Figure 2:**
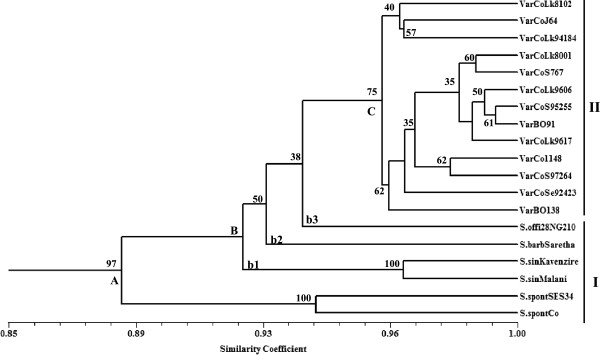
**UPGMA based dendrogram obtained with 515 bands observed with 185 CISP markers.** Dendrogram showing genetic relationships among the clones of *Saccharum* species, and Indian sugarcane varieties, on the basis of Dice’s similarity coefficient, using 185 conserved-intron scanning primer (CISP) markers. The bootstrap values (≥35) are indicated at nodes.

Of the total 337 CISP PPs, 185 have reacted and revealed genetic similarity in the range of 0.88 to 0.99 among 19 genotypes representing 13 varieties of sugarcane (*Saccharum* spp. hybrids) and 4 species (*S. spontaneum, S. officinarum, S. sinense, S. barberi*). When more divergent genotypes were used with first 30 sets of CISP PPs, the pair-wise genetic similarity varied from 0.39 to 0.95, with an average of 0.62 [31]. In general, similarities among varieties were higher than those among the related species (Additional file 3). The generated data using CISP markers were used for determining the phylogenetic relationship among the 13 varieties of sugarcane and 6 genotypes representing 4 species of *Saccharum* (Table 1). The cluster analysis based on UPGMA method grouped these genotypes into three major groups (cluster A, B and C) with bootstrap values ranging from 35 to 100 at different nodes (Figure 2). The CISP markers categorically placed accessions of all four species in class I and all hybrid varieties in class II (Figure 2). Two accessions of *S. spontaneum* clustered together (cluster A) with bootstrap values of 100, indicating a strong affinity to cluster together. The major cluster B, which was also formed by three *Saccharum* species (namely, *S. sinense*, *S. barberi* and *S. officinarum*), formed three distinct sub-clusters (b1, b2 and b3). Similarities between these two major clusters, A and B, comprised 88%. The major cluster C exclusively included all hybrid varieties and was placed close to *S. officinarum*. However, *S. spontaneum* was placed distantly in relation to these varieties, clearly indicating a major genome contribution of *S. officinarum* (>80%) and a minor contribution of *S. spontaneum* (<20%), in synthesis of sugarcane (*Saccharum* spp. hybrids) varieties. The third species which was placed next to this was *S. barberi*, indicating its contribution in generation of sugarcane hybrids, as well. Since the sugarcane varieties used in the present study were selected for sub-tropical Indian regions, *S. officinarum* and *S. barberi*, best suited to sub-tropical regions, were placed close to these varieties. Among the 13 varieties of sugarcane, CoLk8102, CoJ64 and CoLk94184 formed a small group and of these three, the last two are early maturing and high-sugar varieties. The remaining ten varieties formed a separate cluster, and among these the most distinct variety observed was BO138. Mantel’s correlation coefficients between the similarity coefficient and the cophenetic value was fairly high in the marker systems (r = 0.737), indicating very good fit for the clustering pattern, which was also supported by moderate to high bootstrap values (Figure 2).

### Cross-amplification of CISP markers in tropical grasses

**Figure 3:**
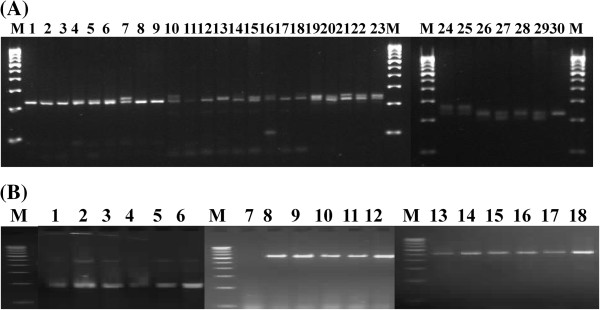
**Transferability of CISP markers of sugarcane.** Transferability of CISP markers in tropical grasses and *Avena sativa* genotypes. (**A**) PCR products (3% high quality agarose gel stained with ethidium bromide) with CISP_SC-01 marker with nine accessions of each of *Chrysopogon fulvus* (Lanes 1–9) and *Sehima nervosum* (lanes 10–18), five accessions of *Heteropogon contortus* (lanes 19–23) and seven accessions of *Dichanthium annulatum* (lanes 24–30). (**B**) PCR products with six genotypes of *Avena sativa* with CISP_SC-05 (lanes 1–6), CISP_SC-13 (lanes 7–12) CISP_SC-19 (lanes 13–18). M = 100 bp DNA ladder as molecular marker.

A set of thirty CISP markers (CISP_SC-1 to −30) [31] were used to find out the cross- transferability potential and polymorphic efficiency in four major tropical grasses, namely, *Dichanthium annulatum*, *Sehima nervosum*, *Heteropogon contortus* and *Chrysopogon fulvus* and six varieties of oat (*Avena sativa*) (Table 1). Of the total 30 PPs, 6 PPs reacted, indicating only 20% cross-transferability in tropical grasses, while 10 PPs reacted with six varieties of forage oat, indicating 33% cross-technical functionality (Figure 3). The number of bands in these species ranged from 1 to 3, of sizes between 150 to 900 bps (Figure 3). When these primer pairs were tested with rice, maize and sorghum, the cross-transferability was 73.7%, 78.9% and 68.4% respectively [30]. This high level of cross-species reactivity was expected, as these markers were designed by merging sugarcane ESTs with homologous ESTs of sorghum a barley, along with genomic sequences of rice. Zeid *et al*. [28] also reported a high rate of cross-transferability (75–91%) of such markers, developed from sorghum and pearl millet ESTs and aligned with the rice genome. In general, the size of the amplified fragments was greater in comparison to EST or genomic-based SSR markers, which can be attributed to the amplification of the intronic region of the genome.

### Molecular basis of polymorphism and functional significance

**Figure 4:**
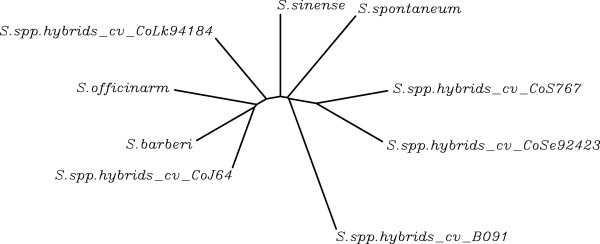
**Dendrogram based on DNA sequences.** Dendrogram generated based on the DNA sequence homology (ClustalW) of four *Saccharum* species and five commercial varieties of sugarcane.

The monomorphic bands observed for the 19 samples, with primer pair CISP_SC-41, were eluted and sequenced, and gave 600 bp sequences. Homology search BlastN, with non-redundant nucleotide database of GenBank, showed that all of these sequences shared homology with Chl a/b binding proteins of rice, maize, sorghum and *Panicum virgatum*, with ~70% identity and an *E*-value of e^-40^. This confirms that the primer-pair amplified its exonic (chla/b binding protein gene) as well as its intronic part. When amplified sequences were aligned together using ClustalW, a low level of similarity (8%) was observed (Additional file 4), indicating a significant sequence divergence among the intronic regions of the 19 samples. Comparing one variety (*Saccharum* spp. hybrid CoLk 94184) with all four species of *Saccharum* revealed more than 150 bps (40%) match. However when all varieties were aligned with sequences of one species, only 10% bases matched, which further indicated either a very high divergence in the sequence of introns or that the primer pair may span a region of introns with high variability. Future studies, such as full-length sequencing of the genes with introns, may provide some insight into these questions. Nevertheless, a high ploidy behavior of the crop may also contribute significantly in this regard. Dendrogram based on sequence homology among varieties and species reflected a reasonable level of closeness among themselves (Figure 4).

The present analysis showed that out of 337 PPs used as representative PPs, of the total 3425 PPs, 185 (55%) CISP markers successfully amplified fragments of different sizes in *Saccharum* species and related genera. Also, a subset of PPs showed 22% reactivity with four major grass species and 33% with oat. Although the designing and development of these primers were based on comparative genome analysis of related species of the grass family, the cross-transferability in tropical grasses was not high. However, our earlier reports have shown a very high transferability of such markers in maize, followed by rice and sorghum, indicating a high level of genome sharing of these species, with sugarcane [31]. Those primers which have not reacted in the present study may probably be due to large intronic region.

In the present study, 41% of the bands scored were polymorphic in nature, depicting a higher level of polymorphism, as reported with SSR markers [9,11,20] for genomic analyses of *Saccharum* spp. and related genera. As in the present study, when introns are used as primary amplicon, a high level of DNA polymorphism is observed even in orphan crops [27]. A maximum of 0.52 polymorphism information content (PIC) and 3.50 marker index (MI) further ensured their use as molecular markers in sugarcane genetic investigations (Additional file 2).

A set of representative CISP markers revealed a convincing and broader genetic diversity estimate, ranging from 0.88 to 0.99, when all bands were taken into account and 0.54 to 0.94, when only polymorphic bands were used with a set of genotypes of sugarcane and species of *Saccharum*. It is very likely that this information will get more intriguing, when large numbers of reported CISPs in the present communication, are considered for such study, especially when one is targeting a large and highly polyploid genome like that of sugarcane. As non-coding regions (introns) evolve much faster than the coding regions (exons) [42], the CISPs being intron-based primers would generate more polymorphism than those developed utilising coding sequences. The present study supports the fact that CISPs facilitate better estimate of genetic diversity as compared to other marker systems reported by several workers in sugarcane [9,12].

## Conclusions

In conclusion, the 3425 CISP markers, generated by comparative genomic analyses of ESTs sequences of sugarcane, sorghum, barley and the whole genome sequence of rice, exhibit utility in genetic analysis of sugarcane, oat and less molecularly characterised tropical grasses, thus constituting a rich pool of resources for genetic diversity analysis and phylogenetic studies. The large number of CISP markers will greatly help in developing the syntenic relationships among the crops of grass family (namely, sugarcane and tropical grasses) and in identifying the much sought trait-linked markers. Further, studies are underway to answer questions related to the amplification of multiple bands by such marker systems, attempting the sequencing of individual bands and isolation of full-length genes. This is more important when a crop like sugarcane is used as template genome, where more than one species is involved in generation of inter-specific sugarcane (*Saccharum* spp. hybrids) cultivars, possessing polyploid and complex genomes.

## Abbreviations

CISP: Conserved-intron scanning primers;EST: Expressed sequence tags;SSR: Simple sequence repeat;PIC: Polymorphism information content;MI: Marker index;PPs: Primer pairs

## Competing interests

The authors declare that they have no competing interests.

## Authors’ contributions

AC designed the study and developed the CISP markers and done the statistical analysis along with SS^1^. RJ and SS^2^ performed the PCR reactions and gel electrophoresis. AKR collected the tropical grasses germplasm and developed *Avena sativa* varieties, also helped in designing the study and writing of manuscript along with AC and SS^1^. All authors read and approved the final manuscript.

## Authors’ information

^1^Division of Plant Physiology and Biochemistry, Indian Institute of Sugarcane Research, Rae Bareli Road, Lucknow 226002, Uttar Pradesh, India. ^2^Division of Crop Improvement, Indian Grassland and Fodder Research Institute, Gwalior Road, Jhansi 284003, Uttar Pradesh, India.

## Supplementary Material

Additional file 1 Details about the total 3425 conserved intron scanning primers (CISP) including the gene annotation with rice homologue. Click here for file

Additional file 2 **Details of 337 CISP primer pairs validated using 13 commercial varieties of sugarcane and 6 accessions of 4 species of *Saccharum *in the present study along with PIC, MI and gene annotation.** Click here for file

Additional file 3 **Dice’s similarity coefficients values of 19 genotypes comprising 13 commercial varieties of sugarcane and 6 accessions of four species of *Saccharum *obtained with 337 CISP markers (CISP_SC-31 to 367).** Click here for file

Additional file 4 **Sequence comparison (intron divergence) of the amplified products obtained with CISP_SC-41 marker using four species of *Saccharum *and five commercial varieties of sugarcane.** Click here for file
