# Supplementary material for: Exploiting EST databases for the development and characterisation of 3425 gene-tagged CISP markers in biofuel crop sugarcane and their transferability in cereals and orphan tropical grasses
Source: BMC Res Notes. 2013 Feb 4;6:47. doi: 10.1186/1756-0500-6-47 (PMC3598963; doi:10.1186/1756-0500-6-47)
Supplement: Additional file 2 — Details of 337 CISP primer pairs validated using 13 commercial varieties of sugarcane and 6 accessions of 4 species of Saccharum in the present study along with PIC, MI and gene annotation. [file 1756-0500-6-47-S2.doc]

**Additional file 2: A list of 337 conserved-intron scanning primers (CISP) of sugarcane having homology with the genes of photosynthesis, sugar metabolism as well as other important characters/traits and used with 19 genotypes comprising four *Saccharum* species and 13 commercial varieties of sugarcane. Along with the sequence of primers both forward and reverse, nature of amplification (P = polymorphic, M = monomorphic, NA = not amplified), number of fragments amplified, polymorphism information content (PIC), and marker index (MI) are described. The details of first 30 primers are given elsewhere [31]**.

| **Name of the primer** | **Rice homologue ID** | **Primer sequence (F/R)**  **5’ to 3’** | **Nature of amplification** | **No. of fragments** | **PIC** | **MI** | **Rice annotation (homology with known gene (s)** |
| --- | --- | --- | --- | --- | --- | --- | --- |
| CISP_SC-31 | 9630_m05324 | GGTGGYCCTGGAAGTGGAAARGGYACACAGTG/ GCWGCACGATTCTCYTCRTTCCTTGG | P(1)  M(1) | 2 | 0.44 | 0.44 | Adenylate kinase, putative |
| CISP_SC-32 | 9630_m05899 | GACAACGACCGCTTCCTCGACGTCGTCGAGC/ ACTTCCAGTTGGTGCTAGGAGRTGTGG | NA | - | - | - | Hypothetical protein |
| CISP_SC-33 | 9635_m00485 | AGGTCCCAGGGCATCCGSTGCGACTACATCGGCTC/ AGCCGATGATCTGGTCSAGGRCGCCG | NA | - | - | - | Photosystem I psaG / psaK, putative |
| CISP_SC-34 | 9634_m02114 | GGCAACTGGGTGMAGGCGCAGGAGTGGGCG/ GATGTCGCCGATGTTGTTGTGCCACGGGTC | M(1)  P(1) | 2 | 0.19 | 0.19 | Clorophyll a/b-binding protein precursor |
| CISP_SC-35 | 9636_m00961 | GACAAGTACGGCGCHAACGTCGACGG/ CTGGTGTTGTAGACGAGGAGGGCGCCGCCG | NA | - | - | - | Photosystem II 10K protein - rice |
| CISP_SC-36 | 9629_m06878 | GAAACATTACAACCCCCTCAGCTGCA/ CATGTAGTTCATGTTTATCCAAATGGCACCTCTCC | P(1)  M(1) | 2 | 0.05 | 0.05 | Alpha-glucosidase I |
| CISP_SC-37 | 9629_m07037 | GCTCTSGCSGTCGCCTCGCCG/ TGCCGTCCCGGGACTTGGTCAGCCGCACGTC | NA | - | - | - | Photosystem II reaction centre W protein, PsbW, putative |
| CISP_SC-38 | 9631_m00172 | ACACTTGGACTGCYGWCTGGATTGAG/ GGTCCCTCCTYTTCTCAAASCCTGGCTGTTTCGGC | P | 1 | 0.05 | 0.05 | Fructosamine kinase |
| CISP_SC-39 | 9630_m05324 | ACACTTGGACTGCYGWCTGGATTGAG/ GCAGCACGATTCTCTTCRTTCCTTGG | P(1)  M(1) | 2 | 0.38 | 0.38 | Adenylate kinase, putative |
| CISP_SC-40 | 9633_m02578 | AGCTTCAACCAGCTGCTCGG/ GGCAGCTGCKCCACARAGMACTCCCC | NA | - | - | - | Chlorophyll synthase, ChlG |
| CISP_SC-41 | 9634_m02114 | CGCCACCACCTCCGGSCGCGTCACCATGTCCGCCG/ GATGTCGCCGATGTTGTTGTGCCACGGGTC | M | 1 | - | - | Chlorophyll a/b-binding protein precursor |
| CISP_SC-42 | 9630_m05324 | GGTCCTGGAAGTGGAAAGGGYACACAGTG/ TGCGYCCCARAAGACGTCTTYCCATCTCTTCCTC | M | 1 | - | - | Adenylate kinase, putative |
| CISP_SC-43 | 9632_m03624 | CCATGGCGCTCGCSTCCACCTCCGCCACCGC/ CCATCGCCCASCGCCCGTGGATCAGCTCSGCCTCC | P(2)  M(1) | 3 | 0.37 | 0.74 | Chlorophyll a/b-binding apoprotein CP24 precursor – maize |
| CISP_SC-44 | 9630_m05324 | GGTGGYCCTGGAAGTGGAAARGGYACACAGTG/ ATAGTACTCAATVACAGGCAAACTRGATTCAAC | P | 2 | 0.34 | 0.68 | Adenylate kinase, putative |
| CISP_SC-45 | 9632_m03624 | GCGCTCGCSTCCACCTCCGCCACCGC/ CCATCGCCCASCGCCCGTGGATCAGCTCSGCCTCC | P(2)  M(1) | 3 | 0.33 | 0.66 | Chlorophyll a/b-binding apoprotein CP24 precursor – maize |
| CISP_SC-46 | 9635_m00485 | CCGTGCCSCAGTTCCAYGGCCTC/ GGCGCCGCAKGCSAGCGTGTCGGCGAG | NA | - | - | - | Photosystem I psaG / psaK, putative |
| CISP_SC-47 | 9631_m00172 | TGCYTGCTTCATGGDGATYTWTGGAG/ GGTCCCTCCTYTTCTCAAASCCTGGCTGTTTCGGC | NA | - | - | - | Fructosamine kinase |
| CISP_SC-48 | 9633_m04546 | TGGCCAAGTACGGSGASAAGAGCGTSTACTTCGAC/ TTAGATCTTGCCGCGYGGKCC | NA | - | - | - | Photosystem I reaction centre subunit VI |
| CISP_SC-49 | 9640_m01293 | GAGATGGCGGYGAAYCTVGAGGACGTGCC/CAGCCCTCARCATATCACCAGTGGCTAAATGGCA | NA | - | - | - | Adenylate kinase a (ec 2.7.4.3) (atp-amp transphosphorylase) |
| CISP_SC-50 | 9630_m02937 | GCCGCCGCCGCCGCCGGAGGAG/ ACCTTGCGGCCTGTCTTCTTCTG | M | 1 | - | - | Heavy-metal-associated domain, putative |
| CISP_SC-51 | 9629_m03017 | CSCTCAAGCTMAGGAAGAAGCG/ GCCTTCTTGTCGTAGGCRCCGCCGACG | P | 1 | 0.19 | 0.19 | Heavy-metal-associated domain, putative |
| CISP_SC-52 | 9632_m03260 | GCTCTGAGAGGAYTRTTCATYATTGACAAGGAGGG/ GACTTYTCCCCWGGYTTCCATCCDGC | NA | - | - | - | Similar to thioredoxin peroxidase |
| CISP_SC-53 | 9629_m04067 | CGGGGAGCAGCCTCATCGGGGCCGCCATCAAGGC/ GTTGGYACACTTTCAAGCTTWGTYTGGAGGATG | M | 1 | - | - | ATP synthase subunit C, putative |
| CISP_SC-54 | 9633_m02330 | AAACTYGGCTGGGCCATTGATGAGG/ GGTAGTAYGCATGYTCCCAGACATC | NA | - | - | - | Probable superoxide dismutase (EC 1.15.1.1) (Mn) precursor – rice |
| CISP_SC-55 | 9633_m02330 | GCGCCTGCACCACCAGAAGCACCACGCCACCTACG/ GAACCAAAATCCTCATCAATGGCCCAGCCAAG | P | 1 | 0.45 | 0.45 | Probable superoxide dismutase (EC 1.15.1.1) (Mn) precursor – rice |
| CISP_SC-56 | 9633_m02578 | GCTGGCCAGGCRTTATTTGGAAC/ AGAHCACCTGRGGAATTGTSAGYCC | P(1)  M(1) | 2 | 0.09 | 0.09 | Chlorophyll synthase, ChlG |
| CISP_SC-57 | 9633_m02578 | CCCACYTCCTSGCCGCCKCCGCCGCC/ GGCAGCTGCKCCACARAGMACTCCCC | NA | - | - | - | Chlorophyll synthase, ChlG |
| CISP_SC-58 | 9636_m00075 | TCATYGCAGTGGATGTTCTTGATGAGAAACTCCAG/ GCRAGCTTSACTATCTGAGGAAGATCTTGCCTGGC | M | 1 | - | - | Alcohol dehydrogenase-like protein |
| CISP_SC-59 | 9631_m06117 | TCGTCTTCGGCGGRTTCGCCGACARGCGCTTCCTC/ TCTGTTTCCTATGGCTGAWGCRGCAGCAAACTC | NA | - | - | - | Putative transcription factor |
| CISP_SC-60 | 9632_m01500 | TGCGGCGGCACCGCCTGCGTCAACTCCTTCGG/ GACGCCHGTCAGCTTGTCRCCRGGCC | P | 1 | 0.33 | 0.33 | Fructose-1,6-bisphosphatase, putative |
| CISP_SC-61 | 9634_m02114 | AGGAGCTGCAGCGCSGTGGGCGTGCC/ GATGTCGCCGATGTTGTTGTGCCACGGGTC | P | 2 | 0.48 | 0.96 | Chlorophyll a/b-binding protein precursor |
| CISP_SC-62 | 9636_m04168 | CMGTGGCTGGTGCTGARKCWGTTGC/ GGCTTCTCAAGGAGYACYTTRAAATAGG | P | 1 | 0.05 | 0.05 | Peroxidase, putative |
| CISP_SC-63 | 9634_m01644 | CCAAGTTYTGGGAGAAGGGCCTYGATGCCACTGAC/ CACATCTCCTGGTCRGAGTTGAGGAGGCCCTCGCC | P | 1 | 0.05 | 0.05 | Peroxidase |
| CISP_SC-64 | 9632_m03703 | GCTGGTGGTGGAGGTSAAGTCSCCGGCRGACAAGC/ CCAGCTCGCCGTCCACCACGCTG | P | 1 | 0.45 | 0.45 | Pathogenesis-related protein Bet v I family |
| CISP_SC-65 | 9633_m03226 | AGAAGATCTTCAAGACCAAGTGCGCSCAGTGCCAC/ TTCTCCTCCCARAYCACAGCC | P | 2 | 0.28 | 0.56 | Cytochrome c [validated] – rice |
| CISP_SC-66 | 9629_m00074 | TTTGGTGCTGAATAYGATGACATTG/ AGCAGCRCTCARAACTCCTGTCATGGTGCCCCC | P | 1 | 0.44 | 0.44 | 1-deoxy-D-xylulose 5-phosphate reductoisomerase |
| CISP_SC-67 | 9631_m01861 | GCYGCRCCGCGCCTSTTCCGYGCCG/ GCCGCSGGCTCCACCTTGATGTGSGACG | P | 1 | 0.19 | 0.19 | Pathogen-related protein |
| CISP_SC-68 | 9629_m06537 | TTGAACYTDGGCTGGTGTGAT/ TCAGCGCCGTGCACTGGCTGATGTTCAGGC | M | 1 | - | - | Leucine Rich Repeat, putative |
| CISP_SC-69 | 9630_m05194 | GCCGARCTYGTGCACTGCCGG/ CAAGAGAAGCTGRACRAAGAAGAGYGC | NA | - | - | - | Light-harvesting complex protein [imported] - Arabidopsis thaliana |
| CISP_SC-70 | 9636_m00961 | GACAWGTACGGCGCHAACGTCGAC/ GCRCTGGTGTTGTAGACGAGGAGGGCGCCGCCG | M | 1 | - | - | Photosystem II 10K protein - rice |
| CISP_SC-71 | 9633_m04082 | TGGACRATGTGGGAGATGATGACTGC/ ACAATAAAAGCTTTAATRTGWCGRATCTTGTC | P | 1 | 0.47 | 0.47 | Proline iminopeptidase |
| CISP_SC-72 | 9635_m00597 | GCTCCGATCCCGGTGCGCCCGCGCCAGTCTTTCGG/ CGGTGGTTGAGGCGGSCSAGCAGCTGCAGCTC | P | 1 | 0.50 | 0.50 | Protein kinase domain |
| CISP_SC-73 | 9629_m03064 | GCTGCAGAAGGYGATGRTGGCGGTGGAYGAGAGCG/CCTTGTCGAGGAGCGCCTGCG | M | 1 | - | - | Universal stress protein family |
| CISP_SC-74 | 9630_m00565 | CCCCCGTCGACGCGCTCCACGGCGACATCGGC/ GCCCRCTTGCCTTSAGTGTMCGCCGGAG | P | 1 | 0.19 | 0.19 | Sugar-phosphate isomerase-like protein - Arabidopsis thaliana |
| CISP_SC-75 | 9640_m01293 | AAGTGCAGCTCCAAGCCCG/ GCAGCCSTCARCATRTCACCAGTGG | P | 1 | 0.05 | 0.05 | Adenylate kinase a (ec 2.7.4.3) (atp-amp transphosphorylase) |
| CISP_SC-76 | 9633_m04546 | CCGGCAGCTCCATCTCCGGCMGSAAGCTCGCCG/ TTAGATCTTGCCGCGYGGKCC | P | 1 | 0.45 | 0.45 | Photosystem I reaction centre subunit VI |
| CISP_SC-77 | 9630_m03636 | AGTGGGTCRTSGASCACAAGCTCCG/ TGGATGATCTTGACGCTRGTCTTCAT | M(5)  P(1) | 6 | 0.05 | 0.05 | Hypoxia induced protein conserved region, putative |
| CISP_SC-78 | 9635_m00485 | GAGGTCCCAGGGCATCCGSTGCGRCTACATCGGC/ TGTTCTTGAGSCCGAGSACGAYGCCGACGCC | NA | - | - | - | Photosystem I psaG / psaK, putative |
| CISP_SC-79 | 9633_m04368 | TGCCATGGCTTCCCACCAGGACMAGGCTAGCTACC/ CGGMGGCCTTCTGCTTGG | NA | - | - | - | LEA protein – rice |
| CISP_SC-80 | 9629_m00716 | GGGAGGTGCGCCACCGGAAGCGGAAACCTCAGG/ GACACCTGGATGCGGTTGCTGCTGC | M(1) | 1 | - | - | Expressed protein |
| CISP_SC-81 | [9630_m00991](http://cgi-www.daimi.au.dk/cgi-chili/GeMprospector/gemalignment?g=birc16&a=/tmp/tmpKaz6G5.dir.chili/Gryder/9629_m03863.aln) | ACTGAAGAAGCTGAACTCAAAATGCTKAG/ TGTTCTTGGARCGRCCRAGCTTTGGTG | M(1) | 1 | - | - | Expressed protein |
| CISP_SC-82 | [9630_m03095](http://cgi-www.daimi.au.dk/cgi-chili/GeMprospector/gemalignment?g=birc16&a=/tmp/tmpKaz6G5.dir.chili/Gryder/9629_m06835.aln) | GAGGCBTACAACGAGAAGAGCA/ CGGAGATCAGATTACAGAGGGCGAGCGTGC | M(1) | 1 | - | - | Expressed protein |
| CISP_SC-83 | [9630_m05206](http://cgi-www.daimi.au.dk/cgi-chili/GeMprospector/gemalignment?g=birc16&a=/tmp/tmpKaz6G5.dir.chili/Gryder/9629_m07321.aln) | CTGCAGAAGCASAACTCRTGGTCGCC/ GCCCACTGCTTCAGCCKCATCTTCACCGTCTCCGG | M(1) | 1 | - | - | Expressed protein |
| CISP_SC-84 | [9629_m07144](http://cgi-www.daimi.au.dk/cgi-chili/GeMprospector/gemalignment?g=birc16&a=/tmp/tmpKaz6G5.dir.chili/Gryder/9630_m01279.aln) | CATGGCCGCGCCGCTCACGGCCGTCCG/ TGTAGTCCACTGTTCGYCGCTGGACAGGCTTCCGC | NA | - | - | - | Hypothetical protein |
| CISP_SC-85 | [9631_m00976](http://cgi-www.daimi.au.dk/cgi-chili/GeMprospector/gemalignment?g=birc16&a=/tmp/tmpKaz6G5.dir.chili/Gryder/9630_m05570.aln) | CCGAGCGGSGCSAAGCTGCTGCAGATCC/ CGCCCACGCCGMGTAGTTSGCC | NA | - | - | - | Hypothetical protein |
| CISP_SC-86 | [9632_m04239](http://cgi-www.daimi.au.dk/cgi-chili/GeMprospector/gemalignment?g=birc16&a=/tmp/tmpKaz6G5.dir.chili/Gryder/9631_m00338.aln) | GCAGCAGCTTCRGTYTTGGCAWCTAAGCT/ GGCCTGAGYTGGAGCCTGAGTATTGCCCC | P(2) | 2 | 0.19 | 0.38 | Hypothetical protein |
| CISP_SC-87 | 9629_m03863 | CTCTGCCAGTGCGAGAMCCTCGACG/ AAACATKCCCAATGGRTGGCATTTCTCCAA | NA | - | - | - | ATP synthase (C/AC39) subunit |
| CISP_SC-88 | 9629_m06835 | CCCCAAGCCGCGCCGCATTCGCTCG/ CCACCKCGGTTCCCTGBAGKTCTSTC | M(1) | 1 | - | - | Similar to glycine-rich RNA-binding protein 2 - rice |
| CISP_SC-89 | 9629_m07321 | GGTCCACCATCGGYATGTGCCTCACCGASACGCT/ CAKGCCACRATCTTCACCTTG | P(1) | 1 | 0.19 | 0.19 | Transcription initiation factor iia gamma chain (tfiia-gamma) |
| CISP_SC-90 | 9630_m01279 | TGTCTTCACTGCYCTTTCTGGTGSTGTTGGAGCTC/ GGATCCCGTCCRACAATHGAGAAVACCTCCTCTG | M(1) | 1 | - | - | Probable citrate synthase [imported] - *Arabidopsis thaliana* |
| CISP_SC-91 | 9630_m05570 | CTCCTCCGCKCCGAGGGSTTCGAGCACTACCGCTG/ CCGATGCTGCTMAGRTCCTTGCAGATCCT | NA | - | - | - | Proliferating cell nuclear antigen (pcna) |
| CISP_SC-92 | 9631_m00338 | TGGCAKCAGGYTTGCAAGTGTTTGGCCAGCC/ AGGTCAGCCARGGTGAAYTCATCRCCAGCCAGG | M(1) | 1 | - | - | Glutathione S-transferase, C-terminal domain |
| CISP_SC-93 | 9631_m00475 | GCCGCCGCCGCTGYCGASATGGGGTTCG/ AGACCCTTMAGACCMATKGTAGCAG | P(2)  M(1) | 3 | 0.36 | 0.72 | HR-like lesion-inducing |
| CISP_SC-94 | 9634_m00341 | AGCTATTTCTTGGCTGGTGCTGC/ GGGGAAGGTCTTBCGGGTGCAMCCRGGGTCCC | P(4) | 4 | 0.36 | 1.44 | 3-phosphoshikimate 1-carboxyvinyltransferase |
| CISP_SC-95 | 9634_m00884 | GGGCTCCTSTCMTTYATGATGGATGATGC/ AGTTGCATYARGGGCAASGCCATCAC | P(2)  M(1) | 3 | 0.1 | 0.2 | probable ubiquitin-conjugating enzyme E2 |
| CISP_SC-96 | 9634_m04829 | CGTCGACGGCCTCGCCAGCTTCCTCG/ GTCTTCTCACAAATRTAAGCATCRCCAATCTG | P(2) | 2 | 0.36 | 0.72 | F-box family protein, putative |
| CISP_SC-97 | 9638_m02672 | CTGTGCTTTGTCTCGCACGCTCTCTCGCCTTCCGC/ TGCATCAGGCARTTCTACYCTAACCTTCCAGACAC | NA | - | - | - | Ubiquitin-conjugating enzyme, putative |
| CISP_SC-98 | 9638_m02901 | GCCGTCATGCGSGACAAGMTCACCGGCCGCCC/ CCASCATAMSCAGCAGGAACACCTCCA | NA | - | - | - | Putative RNA binding protein |
| CISP_SC-99 | 9638_m03184 | CGGTGGCGGAGSTSTGGGAGACGCTGAAGCAGGCG/ TGACGTCRTAGATCTGGCCCWTGATGGCCATGAGG | NA | - | - | - | putative steroid membrane binding protein |
| CISP_SC-100 | 9640_m03693 | CGACTTCGGCGAGCGCAACGGSTACCTCAAGGGCG/ GGGTTCATGGCCACMCCACGCACCTTRGGCCAG | P(1)  M(1) | 2 | 0.39 | 0.39 | 60s ribosomal protein l2 (l8) (ribosomal protein tl2). |
| CISP_SC-101 | 9629_m00940 | CTGCGGCGTCTGCGGCCTCCCCGC/ ACACATTTGCGCTTGTTGCGGACAATCTTCTC | M(3) | 3 | - | - | Density-regulated protein DRP1 |
| CISP_SC-102 | 9629_m01078 | TGGTGGACAGCGCSAACGGGTTCGAGATCCGGCGG/ GCGTCAGCACCGGCGCMGTCATCTCGATCGTCTC | M(1) | 1 | - | - | SOUL heme-binding protein |
| CISP_SC-103 | 9629_m01292 | TCTATAGACATGCCTGCTGGCCCTTC/ ACATCCTYGCGTAACCATAGGTWGGRAGGACATGG | M(1) | 1 | - | - | Histidinol dehydrogenase |
| CISP_SC-104 | 9629_m01510 | CGAGAAGCACASCATGGAGAAGGACATCGCGGAG/ ATCACCCAGAYTTGAATAGYAAGACRGCTTTAG | P(3)  M(1) | 4 | 0.37 | 1.1 | Dynein light chain type 1, putative |
| CISP_SC-105 | 9629_m05429 | GATGGYGGRTTCTTCAAGACWGGTGATAC/ GTATGGAGCAAGYTTATCTTTYGACCMACTTGTC | M(1) | 1 | - | - | AMP-binding enzyme, putative |
| CISP_SC-106 | 9629_m06861 | TGTGAYMATAGAWGATGATTCTGAAAC/ TCCTCCGGCAGGTAGGGCACTTCTTCTGRGCCTG | M(1) | 1 | - | - | RING zinc finger protein, putative |
| CISP_SC-107 | 9629_m07050 | AAGTCCGGCGCCCAGCGCGCCGCC/ CTTGCAYGCCTCCTCRAAGTTG | NA | - | - | - | Esterase D, putative |
| CISP_SC-108 | 9630_m00155 | CCGGCGAGGAGGAGGAGAACGCSGCSGAGCTCAAG/ TGTCGGGGCAGAGRTTGCCGAGSGTGCAKAGCTCG | P(1)  M(3) | 4 | 0.27 | 0.27 | 15.9 kDa subunit of RNA polymerase II |
| CISP_SC-109 | 9630_m04911 | CGCGAGGCSCTCGAGGAGGCCGGCG/ TTTYGCGCTGCTGTTCTCTT | M(1) | 1 | - | - | Protein F10B6.26 [imported] - *Arabidopsis thaliana* |
| CISP_SC-110 | 9630_m05729 | CGAGCGCCATGAACCCGAGAGGCTACCGTAGC/ ACGAGGGTTCCCATCCTCCGACTTGATTTGGTCTC | M(1) | 1 | - | - | Bet1-like snare 1-1 (atbet11) (bet1/sft1-like snare 14a) (atbs14a) |
| CISP_SC-111 | 9630_m05802 | CGMACRGGAGAHATAGTTGTTTTCAA/ GCCCCAGTGCRCCAATYAGCA | P(2) | 2 | 0.47 | 0.95 | Signal peptidase 18 subunit |
| CISP_SC-112 | 9631_m00111 | CTGGGAGGARAGCGAGAARGCKAGAGC/ GCCATCTCCTCCGCCAKGAT | M(1) | 1 | - | - | Remorin, C-terminal region, putative |
| CISP_SC-113 | 9631_m00643 | GGAATGAGYAGTCAATTAGTYAARGCAATGCCAAG/ GCAGAAGAAAGCAADGGKGTAGHCTCTG | P(1) | 1 | 0.43 | 0.43 | Zinc finger, C3HC4 type (RING finger), putative |
| CISP_SC-114 | 9631_m00660 | GGTGGRCAGATGGCGWCGCCSTCSTC/ CAGTGRACRCTAACAATATCAGCACSTGCCTTG | NA | - | - | - | Ribulose-5-phosphate-3-epimerase |
| CISP_SC-115 | 9631_m01049 | CASCCATGGCCGCGGCGTCG/ ACTCTGTAATCYCCTTGCTGAG | NA | - | - | - | Similar to leucine-rich repeat resistance protein-like protein |
| CISP_SC-116 | 9632_m04481 | CTCGCTGCGCTTTTCCGAGTCCTTTTCCCTCCAAG/ ACGGCSGGGTTGASGTGGCCGCC | P(2)  M(2) | 4 | 0.33 | 0.66 | Tonoplast intrinsic protein 2 |
| CISP_SC-117 | 9632_m04664 | CGGCGGCAGGCGGCKGAGACGCT/ AGCCAGTTCCTCCCGCARGATTCYGACCTCTCCC | NA | - | - | - | IQ calmodulin-binding motif, putative |
| CISP_SC-118 | 9632_m05504 | CTGCAAGTGCTGATCTAGC/ TCTCGCCGTGCAGCTTCAAG | NA | - | - | - | Probable translation factor pelota |
| CISP_SC-119 | 9633_m00800 | TGCKGATGGRAGCATATGYTTAGAT/ CGCTTGTTCTCRCTGAAYAKGCGGGC | M(4) | 4 | - | - | Ubiquitin-conjugating enzyme RAD6 |
| CISP_SC-120 | 9633_m00866 | CTTCCACCWATAAGCAAGGCATATGGRACATTGTG/ GCACCAAATATCATCATCCACAAG | P(3)  M(2) | 5 | 0.52 | 1.6 | NADH oxidoreductase, putative |
| CISP_SC-121 | 9633_m01238 | TGCTTCGAGTGGGAGATCTTGATCGTGCT/ CCAGGTAATGGRCCAGGYTCACGAG | M(1) | 1 | - | - | AY168201 putative lactoylglutathione lyase-like protein |
| CISP_SC-122 | 9633_m01995 | ATGCTGCACTGYGGCGCRATCTC/ CCCAGYGGYCTCTGABTCATCCAKGG | NA | - | - | - | Protein F22G5.18 [imported] - *Arabidopsis thaliana* |
| CISP_SC-123 | 9633_m02230 | CCTGTKGCATTTGGTACATCRCATYTGGCCCTTG/ CAACTGCAATAACAAYAGCACCRCAAACTTTTCC | NA | - | - | - | Quinone reductase-like protein - *Arabidopsis thaliana* |
| CISP_SC-124 | 9633_m02263 | CTATAGCTATAGCAGTGCACAACATACCWGAAGGC/ AGGATTGCRCTGATGGCAGCGCTTGG | NA | - | - | - | Similar to 37802-35617 |
| CISP_SC-125 | 9633_m02557 | TGCCATGGASGAGCAGTTCATCCTCCG/ AGATCCAAGAGWGATGCAGGAAAGCT | M(2) | 2 | - | - | TAFII55 protein conserved region |
| CISP_SC-126 | 9633_m03226 | GCGGCGATGGCGTCGTTCTCG/ TTCTCCTCCCARAYCACAGCC | M(1) | 1 | - | - | Cytochrome c [validated] - rice |
| CISP_SC-127 | 9634_m02115 | CCTACAACTCGTGCTCTTTGTCTAGTTGAAACTGG/ GACGAACAATTTCAAKGTCCTCTTTGYCCCTTG | NA | - | - | - | Uncharacterized ACR, YdiU/UPF0061 family, putative |
| CISP_SC-128 | 9635_m03016 | CCTTCAGAAMCASTTGCAAYATGAAGAAATGTG/ GTGCGGAAVGGRTAGTTCAGG | P(1) | 1 | 0.27 | 0.27 | Zn-finger in Ran binding protein and others, putative |
| CISP_SC-129 | 9635_m03272 | AGCCATGGCGTGGAGYGCCGCCGCCG/ GCTGCYGGBGCCTTCTCYGTCTGAACAGCCTCC | P(6)  M(2) | 8 | 0.47 | 2.8 | Endoribonuclease L-PSP, putative |
| CISP_SC-130 | 9635_m03846 | CCCTCGCCGTCAACGCTSTCGCG/ TGARCGCGGATCACCTCRAACGCGTC | M(1) | 1 | - | - | Similar to root border cell-specific protein |
| CISP_SC-131 | 9635_m03971 | TTATAAAAAGCAAACTTGGRATGAACC/ AACCATTCCRTCAGGCTCYTT | NA | - | - | - | Ubiquitin activating enzyme, putative |
| CISP_SC-132 | 9635_m04486 | TCATGGAGATCTACAACCTCGACGACG/ AGTAATTGCTDGTGTAGAATTTCTTCAGG | P(3) | 3 | 0.39 | 1.2 | Complex 1 protein (LYR family), putative |
| CISP_SC-133 | 9635_m04796 | CCCCCGCTCGCGCAGAAGGCCGCCG/ CTTCATCTGYGCCTTCTCRGCAT | NA | - | - | - | Ribosomal protein S8e |
| CISP_SC-134 | 9636_m02317 | GAGGTSGTBGCGCGCAGYGTGCAGGG/ TCATCSAGCACATCTTGCATCTTCC | NA | - | - | - | GMP synthase, C-terminal domain, putative |
| CISP_SC-135 | 9636_m02929 | TCGTSGGSAAGTCCAAGGAGGACGTCTCCCTCCC/ CAGCATTCMACAAGAAGATCYTGTG | NA | - | - |  | Repressor protein |
| CISP_SC-136 | 9636_m04428 | TYGCATTGGGTGAAGTDGTYGGYAC/ ACTTCWGCACTGCACATMGGGC | P(3)  M(3) | 6 | 0.50 | 1.5 | Zinc finger, C3HC4 type (RING finger), putative |
| CISP_SC-137 | 9637_m02104 | ARGGGGAAGGGCAAGCAG/ CAAACTCRGCRGGAGGGACYTTGTCCCC | NA | - | - | - | PPIC-type PPIASE domain, putative |
| CISP_SC-138 | 9638_m02756 | CTGGAAAAGGGCAGTTTGAGATAGCCTTGAAG/ AGCAGCAAGVCCCAAGTGCGGRTTTGCGC | NA | - | - | - | Glutamine synthetase, catalytic domain, putative |
| CISP_SC-139 | 9638_m03806 | TGGCSCCCTCSCTCCTCGCCGCCG/ CTCTCAAAYTTAACAGCAGATTTYGG | NA | - | - | - | Putative chloroplast chaperonin |
| CISP_SC-140 | 9638_m03833 | GCGCGSGCGCGGGAGCTYGGG/ GGAACAACBGGCAGRTTGTCCAC | NA | - | - | - | Putative eukaryotic initiation factor subunit |
| CISP_SC-141 | 9639_m02750 | TGTTCTAGCWGGAGTTGGMAGTTTATCCTTGATGG/ TGYTTGCTTCTCTTYCGGCAG | P(1) | 1 | 0.19 | 0.19 | Ubiquitin activating enzyme - like protein |
| CISP_SC-142 | 9640_m02824 | CGGGTACTGGAAGGCSACRGGSAAGGAGAAGCCGG/ CTCTCCTCTCCCTGCTGCTCTCKCGTTGCT | NA | - | - |  | Similar to NAC domain protein NAC2 |
| CISP_SC-143 | 9640_m03650 | GGAGCGGCGGGGSATCCCGGCGGC/ GCAACAATATCCAAGCACTTCTCTATATCAGG | NA | - | - | - | Probable prefoldin subunit 3 |
| CISP_SC-144 | 9629_m06992 | CACCGSCGCCCACAAGCARCCCSACTTCCTC/ CGGGTAGAAGTGGTGCGACTCCACCTCCAGCCAC | NA | - | - |  | Glutathione S-transferase, C-terminal domain, putative |
| CISP_SC-145 | 9629_m07245 | CCATGATCATCYCCAAGAAGARCCGC/ ACCAGTAGTAGTRCTGCCASGAGAAGGTCTCCCT | P(3) | 3 | 0.27 | 0.77 | Plectin/S10 domain, putative |
| CISP_SC-146 | 9630_m00026 | TCGCCGATGCTCCACAAGAACCTCCGCGCGC/ TTTGGYCCCCATGCAGGTGCATTGAC | NA | - | - | - | AY039615 AT5g61230/maf19_230 |
| CISP_SC-147 | 9630_m04747 | CGGAGATGKCGGAGARGAAGCGCG/ CCATGGCCTTCTGDGCRAACTTCCTGATCTCCTTG | M(1) | 1 | - | - | Ribosomal protein L31e, putative |
| CISP_SC-148 | 9631_m00338 | CTGCAGCAGGTCGGTCATCCAGYACATTGCTCTCG/ GAACRAGCAAAAMSGCCCGGACACAT | NA | - | - | - | Glutathione S-transferase, C-terminal domain |
| CISP_SC-149 | 9631_m00828 | ATGCCTTGACTGMACATGTCAAAGC/ ATCGATCAAGAGCAAACAATATGCACG | NA | - | - | - | CutA1 divalent ion tolerance protein |
| CISP_SC-150 | 9631_m01861 | TCCRCCAACAGCTGGACCCWCGAGATC/ GGCTCCACCTTGATGTGSGACG | NA | - | - | - | Pathogen-related protein |
| CISP_SC-151 | 9631_m02852 | GAGGARACYGAAGAGGAGAAGA/ CTTCTTRATGCCATATCCAACTGG | NA | - | - | - | EF-1 guanine nucleotide exchange domain, putative |
| CISP_SC-152 | 9631_m05300 | TGCTCCAAGTGCCCYGACTTCGACCTCTGCG/ TCYGGRCAAATAAGWGGGAAAGACAGG | NA | - | - | - | Putative transcriptional adaptor |
| CISP_SC-153 | 9631_m05958 | CCGACGCCGCCTTCTTCCGCGACGTCG/ CGTGCTCSCCGATGATGGAMTCCAGCACSGCCTCG | P(3) | 3 | 0.45 | 1.4 | Chalcone isomerase (with alternative splicing products) |
| CISP_SC-154 | 9631_m05968 | AGTCSAARAGGMTGCACCGCTTCAT/ TCATGKTCTTCTGSAAGCCATGAGG | NA | - | - | - | Putative actin depolymerizing factor |
| CISP_SC-155 | 9632_m04779 | GGAGCYGGCAAGGCTGCCGACCTCACC/ CTTGAACTCGTYCCACTCGG | NA | - | - | - | Similar to uridine diphospho-glucose dehydrogenase |
| CISP_SC-156 | 9633_m00512 | CGCSGGSCCGCTCCGCCCCGG/ CTCVCGCGCCASCTCCGGCACGCCC | NA | - | - | - | Rubber elongation factor protein (REF) |
| CISP_SC-157 | 9633_m00858 | ACTCTTCCKTTTGCCACDATGGGTTCTGG/ AACTSGTGGTTTCTCAAGTAYTCTG | P(1)  M(1) | 2 | 0.27 | 0.27 | Proteasome A-type and B-type, putative |
| CISP_SC-158 | 9633_m02330 | AAACTYGGCTGGGCCATTGATGAGG/ GGTAGTAYGCATGYTCCCAGACATC | NA | - | - | - | Probable superoxide dismutase (EC 1.15.1.1) (Mn) precursor - rice |
| CISP_SC-159 | 9633_m03504 | TGACGCCGAAGACCTCGAGCGCGGCGAGCTG/ GGAGCAGCCGAGCGTGATGCCGGCGCCGGAC | M(2) | 2 | - | - | Zinc finger, C3HC4 type (RING finger), putative |
| CISP_SC-160 | 9635_m00839 | GGAAGYGCACATTTCATAAACAAATGYGATAATGG/ GGTTCACAATCGCCCACAAATGACAAACCAAACTG | NA | - | - | - | Thioredoxin, putative |
| CISP_SC-161 | 9635_m04705 | CCATGGGACGAYGARACTGAYATGAAGAAG/ AACTTTAGAYRCAGAACCATTGWAAACTC | NA | - | - | - | EF-1 guanine nucleotide exchange domain, putative |
| CISP_SC-162 | 9638_m02865 | GAAGCTGTTACWGTVCATGCRACTCAGAAG/ GGAAGTAWCCWTTTTCAGAGAARGACATAGCAG | M(3) | 3 | - | - | Putative WD-repeat containing protein |
| CISP_SC-163 | 9629_m00429 | GAGTTCACMTGCACAGCYTTTCGTGAGGAG/ CCTTSAGCTTDGTCATCCACC | NA | - | - | - | Mitochondrial glycoprotein, putative |
| CISP_SC-164 | 9629_m00575 | GGGAAGCYTTRATSCGTGCAAAGCGTG/ GCCCAAGCAGCAGTTCTTGCTGTAGAACC | M(2) | 2 | - | - | Importin alpha-like protein |
| CISP_SC-165 | 9629_m01689 | CAAAATGTGGATACTGGAGCCATTCTYATGCAGGG/ TTGTAGWGCATCATARACTGAAG | NA | - | - | - | Phosphoribosyl-AMP cyclohydrolase, putative |
| CISP_SC-166 | 9629_m03294 | TGCCCYTGTGGGGTGGTCCTKGARAAGGAAG/ AGCATGACYGACCGTCCACARGARTCACATGGTGC | NA | - | - | - | Ubiquitin fusion degradation protein UFD1, putative |
| CISP_SC-167 | 9629_m04177 | AGCTGCTTTGCTAGGATTYGTGGGGGCCCCATGG/ CCACTGTCCAGTCAAGMCCAATAACATCRACTCCT | P(3)  M(3) | 6 | 0.43 | 1.3 | Uroporphyrinogen decarboxylase, putative |
| CISP_SC-168 | 9629_m04690 | CCGGACTCGGTSAAGAAGAGGGAGCG/ TGCATGGCRCCAACCTTCACTGTCTTGTC | P(1)  M(2) | 3 | 0.19 | 0.19 | Ribosomal protein S20, putative |
| CISP_SC-169 | 9629_m05211 | CTTCAKCTAAGATTGCTGC/ CCAAYTTTKGAGTCAACRGCTTGCT | NA | - | - | - | Similar to phosphoesterase |
| CISP_SC-170 | 9629_m05319 | CCCCGCGAGCAGCGMTTCATGTTCG/ AACAAAAGCYCCCATGTCCACACTKGGAGTG | NA | - | - | - | Ribosomal protein L13, putative |
| CISP_SC-171 | 9629_m05395 | GASCAAGAKAAGGTGGAGACTACTTTYAAGCC/ GCATCAGACCAVGCYTTGGCTATTGTGCG | NA | - | - | - | SGS domain, putative |
| CISP_SC-172 | 9629_m05967 | AGAAGAWTCTAATGAAAGGATGTTGGTCTATG/ ATTGAAKCCAACACAWCCATATCC | NA | - | - | - | Protein kinase domain, putative |
| CISP_SC-173 | 9629_m06055 | GGCGGGATGCTSGTGCAGCGCAGGGACGGCG/ CTCTTCTCCTTCCCACGGAAGAAGAGCCTCTG | NA | - | - | - | BAG domain, putative |
| CISP_SC-174 | 9630_m04287 | CTCGCCGCGCAGCAGCACCGCCT/ TCTTYTTGGGTATYTGHTTCTCRTTTAG | M(1) | 1 | - | - | YbaK / prolyl-tRNA synthetases associated domain, putative |
| CISP_SC-175 | 9630_m04933 | CCGSCGCCGGCGAGCCGGACCTCTC/ ACRTTGATAACTTTYYCAGCATCCAA | NA | - | - | - | Senescencs-related protein |
| CISP_SC-176 | 9630_m05098 | TGTTGAAGCYGTTGCTGACTTGAAG/ CTATAACAGCRTCTCCAAATCCAAARCC | P(1) | 1 | 0.27 | 0.27 | Histidyl-tRNA synthetase |
| CISP_SC-177 | 9630_m05162 | CCAAAGTGGATAYCAGCTCAATGGAAGYGCCTATG/ AGTATCTCTTCACGYCTTTGCTGGCGTTCCTC | NA | - | - | - | Peptidase M16 inactive domain, putative |
| CISP_SC-178 | 9630_m05811 | CGMCTTCTCGCCGTCGCAG/ TGATGAARTCGAKGCGGCCGGTGCC | NA | - | - | - | Replication protein A2 |
| CISP_SC-179 | 9631_m00010 | TGAGTCCAGCATTCGMCGGGAAAATGAAGCATTTG/ CTGATTGTASAGACGACCAGAAGCAAAGCTTTGG | M(2) | 2 | - | - | Similar to nucleotide excision repair protein XP-F homolog |
| CISP_SC-180 | 9631_m00660 | CCWGATTTTATCAAGGCAGGTGCTG/ AAAGCTCTGSCCACCAAACCCAGG | P(1)  M(2) | 3 | 0.10 | 0.10 | Ribulose-5-phosphate-3-epimerase |
| CISP_SC-181 | 9631_m00693 | GGGAAGTGCCATTGGGCTATCTATGTCATTCTAC/ TCCCAGTCAGCAATTTCAGA | NA | - | - | - | Nucleoporin PRECOZ, putative |
| CISP_SC-182 | 9631_m00859 | GTCAACTTCTATGCATAYGACASTTACAGAAAGC/ CCATAGAATACAGCRCCAGAGGGTGCCATGC | NA | - | - | - | Mitochondrial carrier protein, putative |
| CISP_SC-183 | 9631_m01078 | TCACAGRGARGCCTTCAGCATGGCTGC/ GCTGCGCTCMATCTGCTTGAAG | NA | - | - | - | Similar to AY088672 transcription factor CRC |
| CISP_SC-184 | 9631_m01079 | TCATCATCTTCTCCGGCCGCGGCCGCCTCTTCGAG/ AGCTCCTTCAWGCTWAGTGGRCCCA | M(1) | 1 | - | - | Similar to MADS box protein MADS1 - rice |
| CISP_SC-185 | 9631_m01764 | TCAACCACCCCTTCTGGTGCTGCAAGAAGCACACC/ CAAGCCTTTTATMAGCTCTCCCTTCATCAG | NA | - | - | - | Protein T6D22.6 [imported] - *Arabidopsis thaliana* |
| CISP_SC-186 | 9631_m01950 | AGCCCATCGTYGGCGCCRTCGCCGG/ ACCTCTATBGCCTTACTCAGCACCT | NA | - | - | - | Enoyl CoA hydratase, putative |
| CISP_SC-187 | 9631_m01975 | AAAGAGGGGGTAAATASAGTTATGTTCCCTG/ GCTTTGTAAGCCAAKAGWGAATGMCCTAATCC | M(2) | 2 | - | - | Adenylosuccinate lyase |
| CISP_SC-188 | 9631_m02217 | TGACATGTTTGGTGAGATGGATGGYTGGGT/ CATCCCTAGTCRGCCAWYTTGTTCA | NA | - | - | - | Similar to TPR-containing protein involved in spermatogenesis TPIS |
| CISP_SC-189 | 9631_m03085 | ATCAATGATAGCATCTATCTTCTTGATGAGAG/ GATTTCCTCTGRGGACCAGCAAGYTGC | NA | - | - | - | Putative ubiquitin conjugation factor |
| CISP_SC-190 | 9631_m03743 | CATGATTGCWGGTGGAGCMGCTGCRAGGCCTTTTG/ GGGAGGYGTGAAATCAATYTCTATYGG | NA | - | - | - | AY099551 lysyl-tRNA synthetase |
| CISP_SC-191 | 9631_m04540 | CTYATAGAATGYAGGGATGTKCAYAA/ GCCATAACCTTCAAAAYAGTTGA | M(2) | 2 | - | - | Putative ABC transporter, 5'-partial |
| CISP_SC-192 | 9631_m04794 | AGYTTCTGGGGGCCTGTTAC/ GTGCTCCKGTARTGCCAGTCYGGTGAA | NA | - | - | - | Putative Acyl-CoA independent ceramide synthase |
| CISP_SC-193 | 9629_m04071 | TGCGGGAYAAGAACGTGATGCAGCTCAAGAAGC/ CCTCCTTCCTTCTTCTSCAGGCGACA | NA | - | - | - | BT004593 At5g11340 |
| CISP_SC-194 | 9629_m04524 | GCGCCTCGGCGCSTGCGAGGACGACTGCTGGATA/ CCYGAGTACTGRGTCTCTAGTATTGACATGAGCTG | NA | - | - | - | Phosphoglycerate mutase family |
| CISP_SC-195 | 9629_m05469 | AGATCATAAGAGGTGGGATGAAGGAATACT/ TCCAGATATCCTACCCTCRCATGGRCAGCATG | NA | - | - | - | Acetamidase/Formamidase family |
| CISP_SC-196 | 9629_m05673 | CCACCGCGTCCGCGTTCGTGTTCAAGGCGGGCGG/ GCGGTTGCAGTTGTCCTTGTTGCCGCTGATG | NA | - | - | - | Plastocyanin-like domain, putative |
| CISP_SC-197 | 9629_m05728 | GTGATCGGCGCGGGGCAGATGGGCTCGGGCATCGC/ ACCTCWTCTGATGTATCAGCYCCWCG | M(2) | 2 | - | - | 3-hydroxyacyl-CoA dehydrogenase, NAD binding domain, putative |
| CISP_SC-198 | 9629_m05729 | ACTGAYGTKGTYTATGGAAACAGGAATGG/ CGCACACGACAGACATTTCCCTAAATTGCATTCTG | NA | - | - | - | HAD superfamily (subfamily IIIA) phosphatase, TIGR01668 |
| CISP_SC-199 | 9629_m05844 | GCCCCCAGCTTCGGCACCGGCACCAGCTCCCGC/ CAGGTTCCATCTCCATAGCTTCAGAACCCTCCATC | P(1)  M(1) | 2 | 0.1 | 0.1 | FAD dependent oxidoreductase |
| CISP_SC-200 | 9629_m06044 | ACAGGGACRAARATGATCCACAAGGG/ GTGCTTGCYTCATGCTCAACACG | P(3)  M(3) | 6 | 0.43 | 1.3 | FeS assembly protein SufB |
| CISP_SC-201 | 9629_m06154 | TCCTACCTCACCGRCAAGATCCGCTCSGCGCGGC/ CTCGTCGATGTGYTGGAAGGHGRCCATCTGC | M(2) | 2 | - | - | ENTH domain, putative |
| CISP_SC-202 | 9629_m06653 | CGGAGGGCTCARCTTGCTCTTCARAAGGG/ CTGATTGAGCACGRGCTTTT | M(1) | 1 | - | - | PspA/IM30 family, putative |
| CISP_SC-203 | 9631_m00255 | GCGGAGRCGACGCTGCRGCTGCTG/ GCCGCGGCGGCGAGGATGGTGGASCCGGTGGAG | M(2) | 2 | - | - | Anthranilate phosphoribosyltransferase |
| CISP_SC-204 | 9631_m00299 | AGCTCTKGCCAAAAAGGATTTYGAGAACYTGAGGC/ CGYGCCAAGCTCCTAGCATATGCATGTTGCTG | P(1) | 1 | 0.11 | 0.11 | Bromodomain, putative |
| CISP_SC-205 | 9631_m00859 | GCTGGWTGCTGTGCTGARGCAGC/ TCAYTCCACTTTCAGGACTATCTTC | P(2)  M(1) | 3 | 0.27 | 0.54 | Mitochondrial carrier protein, putative |
| CISP_SC-206 | 9631_m01007 | TGGCGCTGAGGGGVGTYTGGCAGCTGCAGAAGC/ CCCTTCARGTTAGGATGYTGWCCACGGAC | P(1)  M(4) | 5 | 0.19 | 0.19 | Mitochondrial ribosomal protein L51 / S25 / CI-B8 domain, putative |
| CISP_SC-207 | 9631_m01117 | GGCGACACYACCAACGGCTGCAACTC/ ATGTTTCCCAGGTCRCCCACATGTC | NA | - | - | - | Copper/zinc superoxide dismutase |
| CISP_SC-208 | 9636_m02931 | ACWGTKCCAGGAACCAAGAAACTG/ CTCTGCATRAAGATYGAYTTCTCCAG | NA | - | - | - | AF132115 cytochrome b-561 |
| CISP_SC-209 | 9636_m03729 | GTTTGATCCDGCACTKAGAGARAGACACAT/ ACACATCGCTCHGAYAGTTGATCAAG | M(2) | 2 | - | - | Phosphoglycerate mutase-like protein |
| CISP_SC-210 | 9636_m03805 | CGGGGCGGCCATYGTGCTGGCCTTCCCGCACGAG/ GCTTCYTTAGCTTTHGCYGCCTCCTCA | NA | - | - | - | AY063114 putative 2-nitropropane dioxygenase |
| CISP_SC-211 | 9636_m04092 | TCGGTTGTTGACWGCGCTGCGTGATGCGGAGC/ TTGTTTTCAGCTATCTCTGCTTCT | P(1)  M(3) | 4 | 0.19 | 0.19 | UvrB/uvrC motif, putative |
| CISP_SC-212 | 9636_m04535 | TGCTSTTCGACCAGGCCACCTACGACAAGYTGCTC/ GCCCTGGTGWAKATCTGCTGGCT | M(2) | 2 | - | - | S25 ribosomal protein, putative |
| CISP_SC-213 | 9637_m00337 | CGYTGGAAACTTSTATGCAGCAT/ TGGACGYTCAGTMGATGGTTGTCTGTGCTTCTTGC | NA | - | - | - | Trithorax protein 1, putative |
| CISP_SC-214 | 9637_m00546 | AATGGAGGCAAGCATWTTAACACATTTAATGCCAC/ ATGTCTCGCATKGGAATCCCA | NA | - | - | - | Exonuclease RRP41 [imported] - *Arabidopsis thaliana* |
| CISP_SC-215 | 9637_m00836 | TGGCSRCGCGGAAGCTCTACG/ GTGCTCATCGTGACAAACCC | P(2)  M(5) | 7 | 0.44 | 0.88 | Plastid-specific ribosomal protein 2 precursor |
| CISP_SC-216 | 9637_m01315 | TGCACTCTACAAGCYTGTCAAC/ CAATTTTGTCACCCAATCCATTGTCCAGG | P(2)  M(1) | 3 | 0.27 | 0.54 | Apospory-associated protein C-like protein |
| CISP_SC-217 | 9637_m01882 | CGTACCTGAGCATGGGYGAGGCGCACCGCCG/ TCGCCTCCATYGCCCAMGGMGTCTCCCAG | NA | - | - | - | PCI domain, putative |
| CISP_SC-218 | 9637_m02301 | GAAGCAGCACTTCGAGGAGGCCG/ TCAAGATGCTCMCGGGCCCT | NA | - | - | - | Similar to ABI3-interacting protein 2 |
| CISP_SC-219 | 9637_m02802 | GCGTTCTCCGCGCTACTTCTCGTCCTCCTGCCGCG/ TGTACACSACCTCGCCRTGCT | NA | - | - | - | 149495 putative iojap protein |
| CISP_SC-220 | 9637_m02876 | CTGCYACTCTTGGAATTAARCTTCCATTTCTKGC/ GGCAGCTCCTCYTCTGAGTAGAGGCG | NA | - | - | - | AY113056 AT3g12300/F28J15_117 |
| CISP_SC-221 | 9637_m02940 | CTCCTCCCTCCCGCGSTGCTCMTCCTCCGC/ TGCAGCGACGAVGAGACGCTGGTCATGTTGCAGAG | P(1)  M(2) | 3 | 0.11 | 0.11 | Glyoxalase II |
| CISP_SC-222 | 9638_m00434 | CCMCCTRCACCHTTGCCTTATGATG/ ATGCARCARAGATGRAAATGATGSTC | NA | - | - | - | AC112513 Putative RING-H2 finger protein RHB1a |
| CISP_SC-223 | 9638_m03236 | GCCCTTGATTTTGAAATTGGAGGAGG/ GATAAYTAAYTTTTGMCCAGCACGAT | NA | - | - | - | Putative DnaJ domain containing protein |
| CISP_SC-224 | 9638_m03954 | TCAGCCAGCTCCTCCTCCTCGACGCC/ GGCTGATGSATCATWATCCTGGTGTTGGGCATGGC | P(7) | 7 | 0.5 | 3.5 | Putative Clp protease |
| CISP_SC-225 | 9639_m01054 | CTCTCTTGAACCCTCCGCCGCTCCGAGG/ CGGTGSCCRGCSCGGCCGCCGCTCTG | NA | - | - | - | bZIP family transcription factor, putative |
| CISP_SC-226 | 9639_m01742 | GGGAAGCTAACCARATTCCYARAGTGAAGGTGGC/ AGTGTTGCRGCTCTTGARACWGCATCCA | NA | - | - | - | Survival motor neuron (SMN) interacting protein 1 (SIP1), putative |
| CISP_SC-227 | 9640_m00616 | GCACATCTATTYTCTTCATTTATCTCAGAGGAGGC/ AGAAACATTGCWGGYTCTATATCATGAGG | P(2) | 2 | 0.48 | 0.96 | Nrap protein, putative |
| CISP_SC-228 | 9640_m00630 | CATCTACGACGACCTCCGCCGCAAGGACCCCG/ TCCTGCCACCKCTGMGGCGCYAGCT | M(6) | 6 | - | - | Ssu72-like protein |
| CISP_SC-229 | 9640_m00995 | CGGCAGGGGAAGACGCGGCTKGCCAAG/ GTTCACACACRTTGCTGAAGAAATGRTCYAATATC | M(2) | 2 | - | - | Clathrin assembly protein AP17-like protein |
| CISP_SC-230 | 9640_m03389 | TGATATCCTTGAAGCACCTGGAGCAACTGGAG/ ACTTCTTGATGATGCCCATCATCTCACTTCCAGGG | P(1)  M(3) | 4 | 0.19 | 0.19 | Metalloenzyme superfamily, putative |
| CISP_SC-231 | 9629_m00017 | GCCGCTTCATCSAGRTGCAGGAGGCCTACGAGAC/ TGAATCCTTYGTCRTGCTCCTCCTCTTCAGC | NA | - | - | - | DnaJ domain, putative |
| CISP_SC-232 | 9629_m01316 | CGACGGTGCACGCSGAGTCSGACTTCGAGGTGCG/ TCAGTTCKGGGAGAGGVACMGGAGGGGAAGC | M(5) | 5 | - | - | SOUL heme-binding protein |
| CISP_SC-233 | 9629_m02674 | GACACGTTCTACCTSACGGACGAGCAGCTGCG/ TCTCTCCTRCRTTCCATTCTRTGGAAGACA | M(6) | 6 | - | - | Cyclin, N-terminal domain, putative |
| CISP_SC-234 | 9629_m03513 | TCTCKTCACCTGAYGTATCYCTGGT/ AGTCAGCTATGACTATRCTAATRTTGTCTT | P(2)  M(2) | 4 | 0.42 | 0.84 | Protein phosphatase 2C, putative |
| CISP_SC-235 | 9629_m04041 | CCGCCGCCGAGATMCGCGCCGC/ TGGTCGTCGTCGAGSGGGTCG | NA | - | - | - | DnaJ domain, putative |
| CISP_SC-236 | 9629_m04127 | CCCAAGAAGGTGAATCTGGTAGCMAAGCTGGTTCG/ CTCCTCTTTCCTCTTGCGRGCCCACYTCGGGC | P(1)  M(2) | 3 | 0.34 | 0.34 | Ribosomal protein L22, putative |
| CISP_SC-237 | 9629_m04262 | CGCTCTMCCTCTCCACCTCCTTCCTCCC/ CCCACTTCTTGCGGAACTTCTCGATCTGGCTGTCC | P(3)  M(2) | 5 | 0.45 | 1.40 | Ribosomal protein L31, putative |
| CISP_SC-238 | 9629_m06826 | CTCACTGAYGGATCRGTDTTTGATTCTAGCT/ TTGACGGCGATRAGCTCMGTGTC | P(1)  M(6) | 7 | 0.19 | 0.19 | Peptidyl-prolyl cis-trans isomerase, FKBP-type, putative |
| CISP_SC-239 | 9630_m05652 | CGAGGAGGGCCWTCTTTRGTDTTTGCTCATTC/ TGMTTCAGTAGAWCCAAGAAGAGG | M(3) | 3 | - | - | Lecithin:cholesterol acyltransferase, putative |
| CISP_SC-240 | 9631_m00207 | CGAGAAGAAGAGCCGCAAGGCCATGATGAAGCTCG/ ACRCTGGCYTGSGTCATGACGAGGTCG | M(1) | 1 | - | - | NAC domain, putative |
| CISP_SC-241 | 9631_m03343 | TCGACATGGACCGGGASCAGCGCCC/ CACCAGATCCAGGGCAAGCAGGCCTC | P(1)  M(3) | 4 | 0.19 | 0.19 | Putative ribosomal protein |
| CISP_SC-242 | 9631_m04129 | TGAGTGCKCTTGAGAAGATKACAAGGGG/ CATRTCCATKTCYTCTGCAGTTCTC | P(1)  M(2) | 3 | 0.19 | 0.19 | Putative nucleotide-binding protein |
| CISP_SC-243 | 9631_m04648 | CCGSCTGCAGAAGGAGCTCG/ CCRTTGCTGTAAATGTGYGGATGCAT | M(4) | 4 | - | - | Putative ubiquitin-conjugating enzyme E2 |
| CISP_SC-244 | 9632_m01510 | GGAGCAATCGAAGAATTAGAGAAAGATC/ CGAATTGGATAGCAGCAATAATCAATTTTAGCGCG | P(1)  M(6) | 7 | 0.19 | 0.19 | Ribosomal protein S3 |
| CISP_SC-245 | 9632_m02214 | CCWGGTGAAAGRGTATGGTTTGG/ TTATGACACTCTTGCACCCTG | NA | - | - | - | tRNA binding domain, putative |
| CISP_SC-246 | 9632_m03535 | AGCCGMATGACGCTGCAGCAGATTCTTGCTCGTGC/ AGCCATGCAGCCACCYTGWCCCAGATTTGGCTGC | M(1) | 1 | - | - | Monooxygenase, putative |
| CISP_SC-247 | 9632_m03645 | GACGSCGGCAGCGCGCTC/ CAATGTTCTGTGCTTCTCTGCCYTGCCT | P(3)  M(2) | 5 | 0.43 | 1.3 | Ran-interacting Mob1 protein, putative |
| CISP_SC-248 | 9632_m04332 | AGGACGTCRCCGAGGAGAAGGCCGWCATCCCCGC/ TTTCCACCCTTGCAAGAGC | M(2) | 2 | - | - | Remorin, C-terminal region, putative |
| CISP_SC-249 | 9632_m04525 | GGTGCTGATGATTCWGCCCAGTCBAAGGCTGCTGG/ CTGCTCTCRTGCCAAGCAGAGCT | NA | - | - |  | Pyridoxal-phosphate dependent enzyme |
| CISP_SC-250 | 9632_m04547 | CAGGTGCCCCTCCTGCAGTGCCTCCC/ ACCAAAGTAGTCRTAYTTATTTAACACATAGTC | P(2)  M(1) | 3 | 0.27 | 0.54 | Acyl-CoA thioesterase, putative |
| CISP_SC-251 | 9632_m05045 | TGGTGGTTTGGAGGTGGTACTGATTTGACTCCTTC/ GGTTRAAYTCCACATARCGACCTCT | P(2)  M(1) | 3 | 0.51 | 1.1 | Coproporphyrinogen III oxidase, aerobic, putative |
| CISP_SC-252 | 9632_m05354 | CGTTCTTCGCGCTSCCGGACGAGGAGAAGGCCAAG/ TGGATGAGCAVSCCCAGCTCSGTGA | P(2)  M(2) | 4 | 0.47 | 0.94 | Oxidoreductase, 2OG-Fe(II) oxygenase family, putative |
| CISP_SC-253 | 9632_m05524 | GAGGAAGCTCARGTTCCAYGAG/ TCCATYTGCTTTATRATGTTVACGAGCTT | NA | - | - | - | S4 domain, putative |
| CISP_SC-254 | 9632_m05575 | TGGGGAACTGCCAGTCTTATYGTCCGAGCACC/ ATCTTCTGGTAAAGCCAYCTCTGMGC | NA | - | - | - | X92762 tafazzins protein from Homo sapiens. |
| CISP_SC-255 | 9632_m05615 | AGGTCCRAAGGAGCACCATGCWGCTCAACCTC/ AGGTTGATCATCGAGTTGGCCARCCCGGCTTC | P(1)  M(4) | 5 | 0.52 | 0.52 | UBX domain, putative |
| CISP_SC-256 | 9632_m05817 | TGGGACCAGGATTTKCWCAGTTTGC/ AAYGCACCWGCTGCAGCAGGATC | NA | - | - | - | Ribosomal protein S17, putative |
| CISP_SC-257 | 9633_m00088 | ATGGGAACACATYAAAGATGAAGMTATTAGAAAGG/ ATGTTCCTTTYGAGCAARTAACTCG | P(1)  M(3) | 4 | 0.19 | 0.19 | tRNA pseudouridine synthase B, putative |
| CISP_SC-258 | 9640_m01851 | AGATTCARCAAGTYCTTGTTGAAATYGGTGACAAG/ TCATYACAGRAGTTCCCTGGG | M(3) | 3 | - | - | BC016577 RIKEN cDNA 2700038N03 gene |
| CISP_SC-259 | 9640_m03368 | TCAAAGATTGGCCTGCAGCTGCACAGGAGGG/ CGAATATCTGGAATRTCAAAVAGGACCATTGC | P(1)  M(1) | 2 | 0.34 | 0.34 | Phenylalanyl-tRNA synthetase |
| CISP_SC-260 | 9629_m00778 | ATGCAGAGYAGAGCKGAGGCTCT/ CATCAGCACAAAGAATATCTTCATCATCAGCCACC | M(2) | 2 | - | - | Syntaxin, putative |
| CISP_SC-261 | 9629_m01622 | AAAGATATTAGCTTGAGTGGGGAGCATGTAAGAG/ CTATCHCGKCCTTGYTTTGCACTACC | NA | - | - | - | DNA gyrase, B subunit |
| CISP_SC-262 | 9629_m03225 | CTCCGCCGCGAGGGCTCTCCGCTCGC/ CCCGTASTGCCKCAGCRCGTCGGAG | P(1)  M(3) | 4 | 0.19 | 0.19 | Protoheme IX farnesyltransferase, putative |
| CISP_SC-263 | 9629_m03260 | GATGCCTATGTTGTTGGMAAAGARAAYGCTCCTGG/ AGCCAYTTAACTGARGCMTGAATATCCTTG | NA | - | - | - | Carboxymethylenebutenolidase family, putative |
| CISP_SC-264 | 9629_m03490 | TGTGCAGAAAGTGGTGARGAGAG/ CCCTTTTCCAAAAGAAACGAACAAACTGCACAGTC | P(1) | 1 | 0.1 | 0.1 | Ubiquitin carboxyl-terminal hydrolase, putative |
| CISP_SC-265 | 9629_m03491 | CCTGATCGCCGCCGGKGCCACCGCCGTCTGCTG/ AGTCGAGGTAGCTGAGMGCGTGGGTGG | M(1) | 1 | - | - | F-box domain, putative |
| CISP_SC-266 | 9629_m06141 | AGCCAGGCGAGCCTCCTVCTCCAGAAGCAGCTC/ TTGGGATCHTCRCCAGGBGGATGAAG | NA | - | - | - | Ubiquitin conjugating enzyme, putative |
| CISP_SC-267 | 9629_m06537 | TVGACTTGTGYGGYTGTGTTCTKAT/ TCAGCGCCGTGCACTGGCTGATGTTCAGGC | P(1)  M(4) | 5 | 0.39 | 0.39 | Leucine Rich Repeat, putative |
| CISP_SC-268 | 9631_m02430 | ACTAGCTTTTCTGATCCTGGRACTGT/ GCCACAAAAWAGCGAGWATTTTYCTCCCCAAT | M(1) | 1 | - | - | DHHC zinc finger domain, putative |
| CISP_SC-269 | 9631_m02598 | GATCTGATGCGGCGGATGCCGCCG/ TCATTTGCCTCRAYYTCAAGATTCCTCATC | NA | - | - | - | Putative capping protein beta subunit |
| CISP_SC-270 | 9631_m02691 | GCTCCAGCAGATTTYCGTTTYCCAAC/ ACAGAGGTCCAGGGAAGGTGCC | NA | - | - | - | Cytochrome c oxidase subunit 6b-1 |
| CISP_SC-271 | 9633_m03756 | AGTACAGAGACAGTAGAGCCACCA/ TTGCTGATGTCYCTCTGGAGCAAAG | NA | - | - | - | Acetyltransferase, GNAT family, putative |
| CISP_SC-272 | 9633_m04739 | CGSCGAGGAGGAGGCGGCGGCGAC/ GAGATCTTGCTMGGCGTRTAGCTCTC | NA | - | - | - | Anaphase-promoting complex, subunit 10 (APC10) |
| CISP_SC-273 | 9634_m00526 | GGCTTGTACTATGTTGAYAGTGAGGGYGCAAGGC/ ACCGGGTAGWASTKGTAGTGCAGCTC | NA | - | - | - | Proteasome A-type and B-type, putative |
| CISP_SC-274 | 9634_m00660 | TGGGCCAAGGGYCAYTACACMGAGGG/ AGGCCTTGCGCCGGAACATGGCCGTG | M(2) | 2 | - | - | Similar to 133549 beta-tubulin |
| CISP_SC-275 | 9634_m01014 | GCAATCSGAAGCTCWAGGCTKTCAAC/ CCACCTCCTCCTTGYTGAAAAATCTYAGCARATCC | M(2) | 2 | - | - | Protein T30F21.6, putative |
| CISP_SC-276 | 9634_m01086 | TCCAGAGCCAGGTTCTGGAGGCTG/ TTCTACCAAGYGTCATCATCTCACCAAG | P(2)  M(3) | 5 | 0.34 | 0.68 | NADH dehydrogenase, putative |
| CISP_SC-277 | 9634_m02103 | ACACCRTCAACCTCCACAAGCGCCTCC/ TCCTCRTCGTTCCTCYTGCGGGCAA | M(4) | 4 | - | - | Ribosomal protein L31e, putative |
| CISP_SC-278 | 9634_m02115 | TTTTTTCCGTCTTCTTTCRAATGTCAAAGCAGACC/ **GGYCTGTATGCCCAGGCYGGTGGCA** | M(1) | 1 | - | - | Uncharacterized ACR, YdiU/UPF0061 family, putative |
| CISP_SC-279 | 9634_m04510 | AGGAATTTCCAYGGATGGAATTACCG/ AGATGTYTGAGCTAAAAGCCARAGGTGATAGAACC | P(2)  M(2) | 4 | 0.50 | 1.0 | Rab geranylgeranyl transferase, putative |
| CISP_SC-280 | 9634_m04664 | AGCAGCGSCTCCACTACCGCTCCGARTGGCACCGC/ TCAAGGTCAGCATCTTCATCMTCCCCRCCATCTCC | NA | - | - | - | Zinc finger protein, putative |
| CISP_SC-281 | 9634_m04858 | TTCCAACATGGHGCATTCATACCTGGCTACCC/ ATGGCTGCTTCYTGAGGTGCGCACA | M(5) | 5 | - | - | Acyltransferase, putative |
| CISP_SC-282 | 9635_m00839 | CCGAGGAGGGAGTCGTGATCGCSTGCCAC/ TCMACCTTCAGGAAGACAGC | P(3)  M(3) | 6 | 0.43 | 1.3 | Thioredoxin, putative |
| CISP_SC-283 | 9635_m02960 | AGAGTCGGTGGGCTRTCTTCCAGGAKGCTAG/ GGCACAGCTTTACCAGATTGCTCYCGTTGT | NA | - | - | - | RhoGAP domain, putative |
| CISP_SC-284 | 9635_m03432 | AAGYTGGTTGAAATGATGTGCAGTG/ CATCCAGGATGCTTYTGCATRATGMCAGCAAATGC | P(2)  M(3) | 5 | 0.15 | 0.30 | Acyltransferase, putative |
| CISP_SC-285 | 9635_m03739 | CTCGATGGGCAAGTGTMAATCTWGGGATCTT/ TGCAACTTGCTCTGGCAGCCATG | NA | - | - | - | GTP-ase activating protein for Arf, putative |
| CISP_SC-286 | 9638_m02188 | CTACCATGACAACCATGTSATYCARGACATGCTGG/ AGCTGTGGTTCTCRATCAGCCACTGCTCCAGCC | M(2) | 2 | - | - | Dwarf protein, OSDIM - rice |
| CISP_SC-287 | 9638_m02672 | CCRAGCAAGCGCCGBGAGATGGAC/ CCAATBGABGGRGATTTGTAAGGAT | NA | - | - | - | Ubiquitin-conjugating enzyme, putative |
| CISP_SC-288 | 9638_m03184 | CGGTGGCGGAGCTSTGGGAGACGCTGAAGCAGGCG/ TGACGTCRTAGATCTGGCCCWTGATGKCCATGAGG | P(1)  M(4) | 5 | 0.10 | 0.10 | Putative steroid membrane binding protein |
| CISP_SC-289 | 9638_m03338 | ATGGCCCACGTCMTSGCCGCC/ GATGATGTCGGCGCCRCCGC | P(2)  M(2) | 4 | 0.27 | 0.54 | Putative gamma-lyase |
| CISP_SC-290 | 9638_m03605 | GATGAGCAGCCGMAGCCC/ TCTCTAGCAAAATGYCCWGGACGCTTAC | P(1)  M(3) | 4 | 0.34 | 0.34 | Putative zinc finger protein |
| CISP_SC-291 | 9638_m03751 | TCCTTGGACTGAAGAAGAACACCGGAGGTTTTTGC/ TCTGGCATGATAGCAGAAGCKGAAGARCTCTCTGC | P(2)  M(2) | 4 | 0.11 | 0.22 | putative Myb-related protein |
| CISP_SC-292 | 9640_m01293 | AGTGCAGCTCCAAGCCCGACAAGCGC/ CAGCCCTCARCATATCACCAGTGGCTAAATGGCAC | M(2) | 2 | - | - | Adenylate kinase a (ec 2.7.4.3) (atp-amp transphosphorylase) |
| CISP_SC-293 | 9633_m02323 | ATTGATGTGCGCAACATGTATGAGATACGCATAGG/ TGCCGWGCCCTWGCCCTTTCCTTCTCCTCCTC | P(1)  M(3) | 4 | 0.11 | 0.11 | Rhodanese-like domain, putative |
| CISP_SC-294 | 9633_m02659 | ATGKTGATTTCTTCAARTGGCTTT/ ACAGGTCCAATATCAATCTTGAAWGGGCACTT | NA | - | - | - | Similar to DNA polymerase alpha subunit IV (primase)-like protein |
| CISP_SC-295 | 9633_m02961 | CTTCAATTGGRTCCGTTCCTGCTCCAARGGAG/ ATGAAAYTCATGATKGCATGGAAG | NA | - | - | - | Similar to AT5g45290/K9E15_7 |
| CISP_SC-296 | 9633_m03035 | AGCTYTATGCAGARCGCATYTTCAAG/ CCWTGGASAGCCTGRCTGCTGTASCT | M(1) | 1 | - | - | Cyclin, N-terminal domain, putative |
| CISP_SC-297 | 9634_m00067 | TCGGMTCCAAKACCCTYACCTGGGTCGC/ CTTTCTTCTTGGGCCTCTCCTGCTCCTC | P(3)  M(1) | 4 | 0.37 | 1.1 | Septum site-determining protein MinD |
| CISP_SC-298 | 9634_m00068 | CAAGCAGAAGARGAGGCYAAGATGAAGGARGCTGA/ CGTTTYGGTGTCTTRGTCTCTCCACG | P(3)  M(3) | 6 | 0.31 | 0.93 | Cwf15/Cwc15 cell cycle control protein |
| CISP_SC-299 | 9634_m00122 | GATGTTGCATTCCAYGAYAGGAGRATACC/ CAGGTTYTGCAGAAACTCCTC | P(2)  M(2) | 4 | 0.22 | 0.44 | Phosphorylase family |
| CISP_SC-300 | 9634_m00441 | TGAAGRCAATAGCTKCTGAAGG/ TGGGGGACRAAGCTCCAYTGCTCYGC | M(3) | 3 | - | - | DNA repair protein XRCC1, putative |
| CISP_SC-301 | 9634_m01016 | CCTTCTGGTTATGGAAATTAYACTCATCCAGCTGG/ TGTTGATGCACCATGGGRTGYGGACTTAC | NA | - | - | - | Similar to gb protein |
| CISP_SC-302 | 9634_m03175 | TGCAAACTTWGCCCTGAGAAAGATCC/ CTCATTYTTCATGTCATKGATGCMACTCA | P(1)  M(2) | 3 | 0.19 | 0.19 | Zinc finger in N-recognin, putative |
| CISP_SC-303 | 9634_m04390 | CGCGCGGCCGTGGAGCAGGGTGGTCG/ TGAAGGGCAGTCACCCCTTTGTCWGTGCGCCCAGC | P(1)  M(3) | 4 | 0.39 | 0.39 | tRNA pseudouridine synthase, putative |
| CISP_SC-304 | 9634_m04747 | GAGTACAATCAGCTTGATCCACTCCTTCGTGCAAC/ CAAATTCMGAAATGGCACCRTGTGTTTTC | P(1)  M(1) | 2 | 0.19 | 0.19 | UTP--glucose-1-phosphate uridylyltransferase, putative |
| CISP_SC-305 | 9634_m04883 | CGGCRGGGTWCCTGGCGCGGCG/ TCCGCATCVTCGATRAGCCTATCGATCAC | NA | - | - | - | Complex 1 protein (LYR family), putative |
| CISP_SC-306 | 9635_m00710 | CGYGAGGAYCACACCATYGGCAACA/ CTCAAGCTCCTTGTCTARATCATTG | NA | - | - | - | RNA polymerases L / 13 to 16 kDa subunit, putative |
| CISP_SC-307 | 9629_m01567 | CGTGGAGAATGCTTYGGAAGGATAACRATTGAAGC/ GCCATGTGGTGCTCCATGAAC | NA | - | - | - | Glycosyl transferase, group 1 family protein, putative |
| CISP_SC-308 | 9629_m01743 | CCGTCGGCGACGACAAGGGCTGGTACGACGGCCTC/ CCGYTCTCGCACTGCTCGCCG | M(1) | 1 | - | - | Plastocyanin-like domain, putative |
| CISP_SC-309 | 9629_m03017 | CTCAAGCTMAGGAAGAAGCGRCC/ CCSGCSGGBGCCTTCTTGTCGTAGGC | P(3)  M(1) | 4 | 0.47 | 1.4 | Heavy-metal-associated domain, putative |
| CISP_SC-310 | 9629_m04067 | GGGSCCGCCATCAAGGCSCCYAGGATCAC/ GTTGGYACACTTTCAAGCTTWGTYTGGAGGATG | NA | - | - | - | ATP synthase subunit C, putative |
| CISP_SC-311 | 9629_m04070 | GCAAAATSARGCTAAGAAAGTAAARATTCTTTGG/ AACATCAAARMACCTRTAGAAGATATAATCAGC | P(3)  M(1) | 4 | 0.13 | 0.39 | BAH domain, putative |
| CISP_SC-312 | 9629_m04705 | GAGGAGCAGGASTCSCCGCCGG/ TCATGTTAGATWTCTGRAATCCAGATCT | NA | - | - | - | hTAFII28-like protein conserved region, putative |
| CISP_SC-313 | 9629_m05211 | TGCYGCTGGRAGAGAGAAKGATCATAGTG/ ACTTGGCGCTACAATTCCACATCAGAATAACTAGG | P(1)  M(1) | 2 | 0.19 | 0.19 | Similar to phosphoesterase |
| CISP_SC-314 | 9629_m05524 | CAGGCTCGCCAACATCMTGCCCSCCGGCGCC/ ACCMACACTCTTGAACCAGCGTAT | NA | - | - | - | Mitochondrial glycoprotein |
| CISP_SC-315 | 9629_m05729 | CGCGGTGGCGGCCGTCGCGGCGTCC/ CCCCCTCGATGGCCGCTTCRATCGCCCTCGCATCC | NA | - | - | - | HAD superfamily (subfamily IIIA) phosphatase, TIGR01668 |
| CISP_SC-316 | 9629_m06141 | AGCCAGGCGAGCCTCCTMCTSCAGAAGCAGCTC/ GGATCMTCRCCAGGYGGATGAAGAATAG | NA | - | - | - | Ubiquitin conjugating enzyme, putative |
| CISP_SC-317 | 9629_m06367 | GTCRCAGAGCCCSCGGTCGCC/ GCATTCTTCTCYGGAAGRGGAGGT | P(2)  M(6) | 8 | 0.50 | 1.0 | PX domain, putative |
| CISP_SC-318 | 9629_m06705 | GGCTGCGCRACKCACCCGMGGAGCA/ ATCCRTGGTACAACCAAGAAMGAGGAAGCTGAG | M(2) | 2 | - | - | Helix-loop-helix DNA-binding domain, putative |
| CISP_SC-319 | 9629_m06929 | ATGGGAGGRTTTTGCTGYTGCCT/ AGACAAGTAGGGCAGACATC | NA | - | - | - | Zinc finger, C3HC4 type (RING finger), putative |
| CISP_SC-320 | 9629_m06973 | TGGAGGGAAAACCRTYGCTCCTGCYAAGAGAGGTG/ CTGCCCAATYCTTTCKGTTRCTTTGTCATACA | NA | - | - | - | Acyltransferase, putative |
| CISP_SC-321 | 9630_m00565 | CGGCGGCGTTCGCGCAGGCGC/ GCCCRCTTGCCTTGAGTGTACGCCGGAG | P(2)  M(1) | 3 | 0.19 | 0.38 | Sugar-phosphate isomerase-like protein - *Arabidopsis thaliana* |
| CISP_SC-322 | 9630_m01279 | CCCATGGGTGYCCTTGCCAGYGCAATGAGCAC/ AGCAAGGTGCCTARCAGCAGCTGTMGAGCAG | NA | - | - | - | Probable citrate synthase [imported] - *Arabidopsis thaliana* |
| CISP_SC-323 | 9631_m04797 | AGAAGGTGGARGGGAACCACAAGACCTACTACCAC/ ATGCACCATYGTWAGGCARTCATCCAAGTC | P(3)  M(2) | 5 | 0.40 | 1.2 | Pescadillo-like protein |
| CISP_SC-324 | 9631_m04852 | GCGCTYACAGAGGAWAGRAAGAAGATGCTTACTCC/ ACAAGATTGGCCATYTGAGCAT | NA | - | - | - | Putative MYND finger protein |
| CISP_SC-325 | 9631_m04984 | GGTGACGGAGTTCATCGAGGGGYTGGTGG/ CATCTCCAGAAACRGCAGCKGTCAACA | M(1) | 1 | - | - | DAK1 domain, putative |
| CISP_SC-326 | 9631_m05035 | TGTCACATGAYTGGCCTCTGGG/ CGTGTTATTGCAAGCCATTCTTCATCATACTG | NA | - | - | - | Similar to RNA lariat debranching enzyme - like protein |
| CISP_SC-327 | 9635_m01542 | CAGCGAGACGGAGGAGGAGCTCAAGCGCGAGATCG/ GCATHGCYCTGAAAACAGGAGA | P(3) | 3 | 0.34 | 1.1 | BTB/POZ domain, putative |
| CISP_SC-328 | 9635_m03846 | GATGGTTCCCCCATATTAGCAGTKAGTAGTTTAGC/ CCATATACAGTRATYACAGTATCYGTTCTGTCCTC | P(2)  M(2) | 4 | 0.29 | 0.58 | Similar to root border cell-specific protein |
| CISP_SC-329 | 9635_m04800 | GACGACAGCGASCACAGC/ GWAGCCATGGCTGCCSACSACSAGC | NA | - | - | - | Universal stress protein family |
| CISP_SC-330 | 9636_m00367 | TCKAAGGGRTTTGGCTTYGTAAGGT/ ATTCMGCVAAAATAACCCATCCATC | NA | - | - | - | RNA recognition motif. (a.k.a. RRM, RBD, or RNP domain), putative |
| CISP_SC-331 | 9636_m00770 | TTGATAAAGTGGGAYAARGCCAAGGTTGG/ CAATCAGYACAAATAAGCCGTGCAGG | NA | - | - | - | mRNA capping enzyme, large subunit |
| CISP_SC-332 | 9636_m01506 | CGCACGCGCCTCTCCTTCGAGGCCGC/ GGATGTTGCCCTGGCCCATCRCCTGCA | P(1)  M(3) | 4 | 0.34 | 0.34 | Aspartate carbamoyltransferase |
| CISP_SC-333 | 9640_m00483 | TTGCCAATCTATCAACTCDGCT/ AGTGCCCCCARACGRTCTCCAAAACTYAGCTGCAC | P(4)  M(2) | 6 | 0.29 | 1.16 | ACT domain, putative |
| CISP_SC-334 | 9640_m03693 | CTACTGCGGMCGCCGCGCCACGCTCTCC/ GGGTTCATGGCCACACCACGCACCTTAGG | P(1)  M(3) | 4 | 0.27 | 0.27 | 60s ribosomal protein l2 (l8) (ribosomal protein tl2). |
| CISP_SC-335 | 9640_m04033 | GCAGGATGCTCCATCAACTGTAAATGTGATGATTG/ CTCCTGTTGGAATGAAGACTCYCTGGAYACTGGAG | NA | - | - | - | DNA binding protein, putative |
| CISP_SC-336 | 9629_m00429 | AAGAAGGGSTCCGGSGCCGGG/ TGGTGATCTCCTCACGAAARGCTGTGCA | NA | - | - | - | Mitochondrial glycoprotein, putative |
| CISP_SC-337 | 9629_m00929 | GCGTGCGCATGTCCACCGAGGGCCAGGCCGTGCAG/ GCTTCGCCTTGATSGACTCYGTCA | NA | - | - | - | IQ calmodulin-binding motif, putative |
| CISP_SC-338 | 9629_m01547 | CCTCTTCGACTAYTACATCCGCACCGACGAGCGCG/ ACATTCCTRAATGCTTCCCARCCYTCTTC | P(1)  M(1) | 2 | 0.19 | 0.19 | PurA ssDNA and RNA-binding protein |
| CISP_SC-339 | 9629_m01622 | CAYTACTGCTATGATGAKGCTGA/ ACACAACRTTGGCCTCWRCAGCATCTTC | M(3) | 3 | - | - | DNA gyrase, B subunit |
| CISP_SC-340 | 9629_m01776 | CATCTTTGGCKGGTCTTCCTGTTAGGC/ ACTCGTTAAGCTCGTCCAGGATGAGTTGGTGCTG | P(2)  M(7) | 9 | 0.27 | 0.54 | Helix-loop-helix DNA-binding domain, putative |
| CISP_SC-341 | 9629_m03017 | GCCGGCCGGCTTCATCAATC/ GCCTTCTTGTCGTAGGCRCCGCCGACG | M(2) | 2 | - | - | Heavy-metal-associated domain, putative |
| CISP_SC-342 | 9629_m04590 | GAGGARGTGAAYATGTACATYGAGCTCCCCAAG/ CCTTGSCCTTKTATTGGAGATGMCCATGTTTTCCC | NA | - | - | - | Nuclear movement protein, putative |
| CISP_SC-343 | 9629_m05728 | GTGATCGGCGCGGGGCAGAKGGGCTCGGGCATCGC/ ACCTCWTYTGATGTATCAGCYCCWCG | NA | - | - | - | 3-hydroxyacyl-CoA dehydrogenase, NAD binding domain, putative |
| CISP_SC-344 | 9629_m06501 | CTTCCGGGACCCGGATGCGGCGATGAG/ AGGTGGCVTGMGAAGGAAAAGCTGCGC | P(1)  M(1) | 2 | 0.19 | 0.19 | RNA recognition motif. (a.k.a. RRM, RBD, or RNP domain), putative |
| CISP_SC-345 | 9629_m06835 | ATGGCGGCCTTCAACAAGC/ TAACAAAGCCAAABCCTCTTGAC | NA | - | - | - | Similar to glycine-rich RNA-binding protein 2 - rice |
| CISP_SC-346 | 9629_m07098 | AAGCTTGTGGTTCTYGGWATCCCHTGGGATG/ GGAGTVGCTATCTTCACTT | P(3)  M(1) | 4 | 0.34 | 1.1 | RNA recognition motif. (a.k.a. RRM, RBD, or RNP domain), putative |
| CISP_SC-347 | 9630_m00025 | ACTGGAGTACAGRGTCATCGGSGACATGATGAACC/ TTCTCCTCRATCTTCTTYTCRATAG | NA | - | - | - | Ribosomal protein S5, C-terminal domain, putative |
| CISP_SC-348 | 9630_m01295 | ATTGACTTGTCRGACAATGAGATTGTCAAGCTTG/ TCACTTTCTTGAARTCCAGCAAMCGCARATGCTTC | P(3)  M(2) | 5 | 0.29 | 0.87 | U2 snRNP protein A'' - *Arabidopsis thaliana* |
| CISP_SC-349 | 9630_m02270 | CTTCGCTTCCTAAACCCTAGCAATGGCGGCGGCG/ AGGACGGCGCMGCAGCCRAGC | P(2)  M(2) | 4 | 0.27 | 0.54 | Diaminopimelate decarboxylase, putative |
| CISP_SC-350 | 9630_m02953 | TGTCTACCTGCTTTCCAAGTGCTGAARCAACTC/ ATCTTAGAAAAGTTYGCAAAGATRACATTAGCTCC | NA | - | - | - | DNA polymerase family B, exonuclease domain, putative |
| CISP_SC-351 | 9630_m04726 | CAGCTSCTCGRCGCCGGCGTCCGC/ CCTTCCTGTCAGGAATRTCATCTAAAAGTCCCATG | P(3)  M(1) | 4 | 0.29 | 0.87 | Probable steroid dehydrogenase [imported] - *Arabidopsis thaliana* |
| CISP_SC-352 | 9630_m05162 | ATGGATATATGAYATGGACCCTTTYGAGCCTCTG/ GCTTTACTTTGRGAAACAAACTGCTTGAACCTCTG | NA | - | - | - | Peptidase M16 inactive domain, putative |
| CISP_SC-353 | 9631_m00111 | ACGACGACTCCAAGGCYATCGTC/ GCTCTMGCYTTCTCGCTYTCCTCCCAGGC | NA | - | - | - | Remorin, C-terminal region, putative |
| CISP_SC-354 | 9631_m02691 | GCTGCTGCGCCCATGGCCGCSGAAGCCAAG/ TCAACATAGCGTGTGAAACAGTGCCT | NA | - | - | - | Cytochrome c oxidase subunit 6b-1 |
| CISP_SC-355 | 9631_m03133 | TTCCTCTCAAGTGTYATGATCAATGCCAC/ TTTTTGGATCAATTTTYTCAAARAAWACTCTCCT | NA | - | - | - | Putative mechanosensitive ion channel protein |
| CISP_SC-356 | 9631_m03866 | GGCAAGAAGCTCATTCAGATMGAYGTGAGCTC/ GCAAGWGTTATGAGCCTATGRCTRTCCATTG | P(1)  M(3) | 4 | 0.27 | 0.27 | Putative polyketide synthase |
| CISP_SC-357 | 9631_m04540 | GGTGGYATGAAAAAGCGYGTWGCTCT/ ATTGTGCTATGYTGATGRGTSACAAC | NA | - | - | - | Putative ABC transporter, 5'-partial |
| CISP_SC-358 | 9631_m04648 | GACTCRTGGTCMCCRGCGATGACBGT/ CTCCCRTTGCGRCAGTTCCTRACATAGCG | P(3) | 3 | 0.48 | 1.45 | Putative ubiquitin-conjugating enzyme E2 |
| CISP_SC-359 | 9631_m04766 | GTYGGCCGCGACTTCSCRGTGGWGGC/ GTGAARTTGGCGGYGGCGGCGG | NA | - | - | - | 20S proteasome beta 4 subunit |
| CISP_SC-360 | 9637_m03014 | CGGCGCGGCGTTGGGATGCGGACGATCTGCGA/ TGCCGMCTAGCAAGCTTGTTACACA | NA | - | - | - | B-box zinc finger, putative |
| CISP_SC-361 | 9637_m03273 | GGGCACCGCGCSAACGCGG/ GTCGTTGSCGTTGGCHTCCTTCTG | NA | - | - | - | Signal peptidase protein-like protein |
| CISP_SC-362 | 9638_m02598 | CAGATTCGTTATAGYAGGAGYACAAGCAGGG/ CCACCGCTTCGCAAAGCTCYTCCTCA | NA | - | - | - | F-box domain, putative |
| CISP_SC-363 | 9639_m02954 | AGCAGKCMTACATCTGCCGBGACTG/ AGCTGCTCCTTCCTHGCCTT | M(3) | 3 | - | - | Rubredoxin, putative |
| CISP_SC-364 | 9640_m00695 | CGCGCAACGTCAGRGCSAGCGTCGAGGG/ AGGGCAGCAAGCTCCCCYTT | NA | - | - | - | Glutaredoxin, putative |
| CISP_SC-365 | 9640_m03037 | CGTAAGACAATTGCAATGCTCAGCCAGG/ TCAACAACCCCATCRGCACCAACAAAYACCATGTC | NA | - | - | - | Initiation factor 2 subunit family, putative |
| CISP_SC-366 | 9640_m03689 | GCCGCCKCGTTCCTCGAGCGCCGCGAG/ GCRAAGCAACGRACTCCATTRCCACA | NA | - | - | - | Diaminopimelate epimerase, putative |
| CISP_SC-367 | 9640_m04000 | TGGAGGARGTATATGAYGCYTTGGCTG/ CGATAAAGGTGGAAACCATCCATKGGAAGCAT | NA | - | - | - | Phosphoribulokinase / Uridine kinase family, putative |
