# Supplementary material for: Exploiting EST databases for the development and characterisation of 3425 gene-tagged CISP markers in biofuel crop sugarcane and their transferability in cereals and orphan tropical grasses
Source: BMC Res Notes. 2013 Feb 4;6:47. doi: 10.1186/1756-0500-6-47 (PMC3598963; doi:10.1186/1756-0500-6-47)
Supplement: Additional file 3 — Dice’s similarity coefficients values of 19 genotypes comprising 13 commercial varieties of sugarcane and 6 accessions of four species of Saccharum obtained with 337 CISP markers (CISP_SC-31 to 367). [file 1756-0500-6-47-S3.doc]

Additional file 3: Dice’s similarity coefficients of the 19 genotypes constituting 13 commercial sugarcane varieties and four species of *Saccharum* obtained with 515 CISP bands.

|  | Var CoLk 8102 | Var CoLk 8001 | VarCoS 767 | Var CoLk 9606 | Var CoLk 9617 | Var CoS 95255 | Var BO91 | Var CoJ  64 | Var Co 1148 | Var CoS 97264 | Var CoSe 92423 | Var CoLk94184 | S.spo.  SES  34 | S.spoCo | S.barSare-tha | S.offi.28NG210 | S.sin.Kave-nzire | S.sin.Mala-ni | Var BO  138 |
| --- | --- | --- | --- | --- | --- | --- | --- | --- | --- | --- | --- | --- | --- | --- | --- | --- | --- | --- | --- |
| Var. CoLk8102 | 1.00 |  |  |  |  |  |  |  |  |  |  |  |  |  |  |  |  |  |  |
| Var. CoLk8001 | 0.96 | 1.00 |  |  |  |  |  |  |  |  |  |  |  |  |  |  |  |  |  |
| Var. CoS767 | 0.96 | 0.99 | 1.00 |  |  |  |  |  |  |  |  |  |  |  |  |  |  |  |  |
| Var. CoLk9606 | 0.96 | 0.98 | 0.98 | 1.00 |  |  |  |  |  |  |  |  |  |  |  |  |  |  |  |
| Var. CoLk9617 | 0.97 | 0.98 | 0.98 | 0.99 | 1.00 |  |  |  |  |  |  |  |  |  |  |  |  |  |  |
| Var. CoS95255 | 0.97 | 0.99 | 0.99 | 0.99 | 0.99 | 1.00 |  |  |  |  |  |  |  |  |  |  |  |  |  |
| Var. BO91 | 0.97 | 0.98 | 0.98 | 0.99 | 0.98 | 0.99 | 1.00 |  |  |  |  |  |  |  |  |  |  |  |  |
| Var. CoJ64 | 0.97 | 0.96 | 0.96 | 0.96 | 0.96 | 0.96 | 0.96 | 1.00 |  |  |  |  |  |  |  |  |  |  |  |
| Var. Co1148 | 0.96 | 0.97 | 0.97 | 0.99 | 0.97 | 0.98 | 0.97 | 0.96 | 1.00 |  |  |  |  |  |  |  |  |  |  |
| Var. CoS97264 | 0.96 | 0.97 | 0.96 | 0.97 | 0.97 | 0.97 | 0.97 | 0.95 | 0.98 | 1.00 |  |  |  |  |  |  |  |  |  |
| Var. CoSe92423 | 0.96 | 0.96 | 0.96 | 0.97 | 0.97 | 0.97 | 0.97 | 0.94 | 0.96 | 0.97 | 1.00 |  |  |  |  |  |  |  |  |
| Var. CoLk94184 | 0.97 | 0.97 | 0.96 | 0.96 | 0.96 | 0.96 | 0.96 | 0.97 | 0.96 | 0.96 | 0.95 | 1.00 |  |  |  |  |  |  |  |
| *S. spontaneum* SES34 | 0.89 | 0.90 | 0.89 | 0.89 | 0.89 | 0.89 | 0.90 | 0.89 | 0.89 | 0.89 | 0.89 | 0.90 | 1.00 |  |  |  |  |  |  |
| *S. spontaneum* Co | 0.88 | 0.89 | 0.88 | 0.89 | 0.88 | 0.89 | 0.89 | 0.89 | 0.88 | 0.89 | 0.88 | 0.89 | 0.94 | 1.00 |  |  |  |  |  |
| *S. barberi* Saretha | 0.92 | 0.93 | 0.93 | 0.93 | 0.93 | 0.93 | 0.94 | 0.92 | 0.92 | 0.92 | 0.92 | 0.92 | 0.88 | 0.87 | 1.00 |  |  |  |  |
| *S. officinarum* 28NG210 | 0.93 | 0.94 | 0.94 | 0.95 | 0.94 | 0.95 | 0.94 | 0.93 | 0.94 | 0.93 | 0.93 | 0.94 | 0.88 | 0.89 | 0.93 | 1.00 |  |  |  |
| *S. sinense* Kavenzire | 0.92 | 0.91 | 0.92 | 0.92 | 0.93 | 0.93 | 0.92 | 0.92 | 0.92 | 0.92 | 0.91 | 0.92 | 0.87 | 0.85 | 0.91 | 0.92 | 1.00 |  |  |
| *S. sinense*  Malani | 0.92 | 0.92 | 0.92 | 0.92 | 0.93 | 0.93 | 0.92 | 0.91 | 0.92 | 0.92 | 0.92 | 0.92 | 0.87 | 0.85 | 0.91 | 0.92 | 0.97 | 1.00 |  |
| Var. BO138 | 0.95 | 0.96 | 0.96 | 0.96 | 0.97 | 0.97 | 0.97 | 0.95 | 0.96 | 0.95 | 0.96 | 0.95 | 0.88 | 0.88 | 0.93 | 0.93 | 0.92 | 0.93 | 1.00 |
